# Priority indicators for evaluating the impact of field epidemiology training programs – results of a global modified Delphi study

**DOI:** 10.1186/s12889-025-21816-2

**Published:** 2025-02-17

**Authors:** James A. Flint, Tambri Housen, Martyn D. Kirk, David N. Durrheim

**Affiliations:** 1 University of Newcastle, Newcastle, NSW Australia; 2 Australian National University, Canberra, ACT Australia

**Keywords:** Field epidemiology training programs, Impact evaluation, Delphi method, Public health workforce, Training indicators

## Abstract

**Background:**

Field Epidemiology Training Programs (FETPs) aim to develop a skilled public health workforce through applied competency-based learning. With 98 programs globally and over 20,000 graduates, these programs play a crucial role in disease preparedness and response activities around the world. Despite their importance, there have been few published evaluations. This paper presents the results of a consensus-building process to develop a preferred array of indicators for evaluating the outputs, outcomes, and impacts of FETPs.

**Methods:**

We conducted a modified Delphi study to reach consensus on preferred evaluation indicators for FETPs. An initial list of evaluation indicators were identified from literature reviews and consultations with impact evaluation experts and FETP professionals. A modified Delphi process was subsequently employed, involving two rounds of surveys and a final expert review meeting, to reach consensus on indicators. The Delphi panel included 23 experts representing diverse global regions and FETP roles.

**Results:**

Consensus was reached to include 134 evaluation indicators in the final impact evaluation framework. These indicators were grouped as output, outcome, and impact indicators.

**Conclusions:**

This study presents the first FETP impact evaluation framework with a comprehensive list of evaluation indicators for FETPs. This list of indicators is intended as a resource to promote and enhance the evaluation of FETPs and thus improve these important training programs which aim to strengthen national, regional and global health security.

**Supplementary Information:**

The online version contains supplementary material available at 10.1186/s12889-025-21816-2.

## Background

Field Epidemiology Training Programs (FETPs) are competency-based training programs designed to develop a skilled public health workforce capable of conducting disease surveillance, responding to acute public health threats, and strengthening health systems based on scientific evidence [1]. These workforce development programs adopt a work integrated learning model, where trainees spend most of their time in their workplace applying skills acquired during face-to-face workshops [2]. FETPs involve a combination of classroom instruction, mentorship, and on-the-job training with an emphasis on practical field experience. They aim to provide a critical mass of competent health workers to respond to acute public health issues and strengthen health systems [3]. Globally, there are 98 FETPs and over 20,000 graduates [4]. Many countries have adopted a three-tiered training approach to train field epidemiologists [5]. These tiers are often referred to as Frontline (basic), Intermediate and Advanced [6, 7]. In addition, there are specialty track FETPs targeting specific audiences, such as laboratory scientists (Field Epidemiology and Laboratory Training Programs, FELTP) or veterinarians (Field Epidemiology Training Programs for Veterinarians, FETPV) [8–10]. There are also programs that focus on specific areas of practice, such as One Health or non-communicable diseases [11, 12]. 

FETPs have become increasingly recognised in national, regional, and global preparedness and response mechanisms to prepare for and counter health security threats. The International Health Regulations (IHR) include explicit targets for the number of trained field epidemiologists for a given population [13, 14]. Surprisingly, given the large number of FETPs worldwide and their importance in strengthening health systems and enhancing health security, relatively few evaluations of these programs have been published [15]. Those that have been published largely concentrate on program processes and outputs, with some also assessing short- or medium-term outcomes. Very few have focused on impact [16, 17]. As a result, there is little direct evidence of the impacts attributable to FETPs. Understanding program impact is critical for optimising training models, curricula and delivery methods, and ensuring that programs remain responsive to country priorities and needs. With international development agencies placing increasing emphasis on development effectiveness and impact [18], there is also a growing need for program directors and faculty to evaluate and report on the impact of their FETPs.

**Fig. 1:**
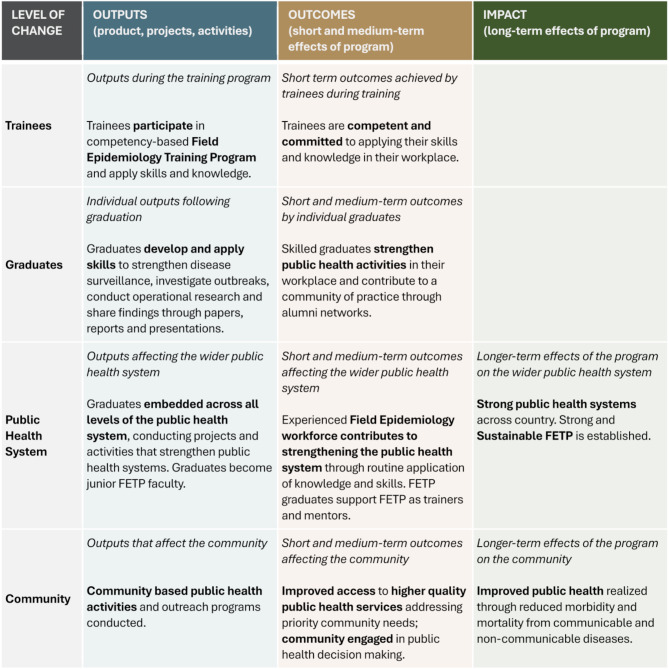
High-level field epidemiology training program impact evaluation framework

To support the evaluation of FETPs, we developed an impact evaluation framework and implementation guide [19]. The impact evaluation framework follows a high-level program theory focusing on change at the trainee, graduate, health system and community levels (Fig. 1). A critical companion to this framework is a comprehensive list of evaluation indicators covering program outputs, outcomes, and impacts. These indicators are measurable elements that can be used to evaluate an FETP’s success. This paper reports on a consensus-building process to refine and prioritise these indicators to support evaluations of frontline, intermediate and advanced FETPs.

## Methods

### Study aim

The overall aim of this study was to establish consensus on a comprehensive set of indicators for evaluating all three tiers of FETP. These indicators form part of an impact evaluation framework designed to support FETP practitioners undertaking impact evaluations.

### Study design

We adopted a two-stage consensus-building approach to compile a list of evaluation indicators for each level of change in the impact evaluation framework (Fig. 1). The impact framework and evaluation indicators were designed for use by regular (non-specialty track) Frontline, Intermediate and Advanced FETPs. The first stage of developing the evaluation indicators has been previously described [19]. In brief, it included a desktop review of the literature, consultations with FETP and evaluation experts, and a review of theory of change documents for FETPs in Papua New Guinea and the Solomon Islands. Once an initial list of evaluation indicators was compiled, input was sought through a structured in-person review process at the Training Programs in Epidemiology and Public Health Interventions Network (TEPHINET) Global Scientific Conference in Panama from September 4–9, 2022, involving 27 professionals representing FETPs from 12 countries. TEPHINET is the global network of Field Epidemiology Training Programs [4]. This review process resulted in several indicator modifications and additions. The framework and indicators were then used to conduct impact evaluations of FETPs in Papua New Guinea and Canada. During this process, further indicators were added or modified. The study was funded as part of a grant from the Australian governments’ Department of Foreign Affairs and Trade. Ethical approval was obtained from the Human Research Ethics Committee at the University of Newcastle.

This paper focuses on the second stage of consensus-building using a modified Delphi process to review and select indicators for use during FETP evaluations. A Delphi study is a widely used method for achieving consensus among a panel of experts on a specific topic. It involves multiple rounds of anonymous surveys, with feedback provided after each round, allowing participant to refine their views until consensus is reached [20, 21]. Delphi studies have been used to support public health practice on numerous occasions [22–24], including selecting training evaluation indicators [25]. One of the challenges associated with Delphi studies is the attrition of the expert panel over successive rounds [26]. Given the large number of indicators under review in this study and the relatively small pool of global FETP experts available for the panel, we adopted a modified Delphi technique to build consensus with minimal attrition by using two rounds of surveys followed by an expert review virtual meeting [25, 27]. 

### Delphi expert panel

We invited experts to participate as Delphi panel members at the Bi-regional TEPHINET Scientific Conference held in Canberra, Australia, from September 12–15, 2023. The impact evaluation framework and the Delphi process were presented to FETP Directors and FETP experts at the conference. The approximately 40 people attending the presentation were invited to express their interest in being a Delphi panel member; each person was given a card with information on the Delphi study and a QR code linking to an online form where they could express their interest and provide contact details. Additional experts were identified through referrals from leaders within the global FETP community, allowing invitations to reach prospective panellists from all regions of the world. To be eligible for the expert panel, participants must have held senior leadership roles in their national FETP program or at the regional or global level in field epidemiology workforce. To avoid any one country being over-represented, a maximum of 4 experts per country were included on the panel, based on a first-come approach. As English is commonly used by the global FETP community, the Delphi process was conducted in English.

### Web-Delphi survey

The 140 indicators compiled during phase one were included in a web-Delphi survey. The questionnaire used to develop the web-Delphi is included as an additional file (Additional file 1). The Delphi survey was administered using Welphi, an online platform specialising in Delphi processes [28]. The questionnaire was pre-tested by FETP professionals working with the University of Newcastle’s Field Epidemiology in Action team to check for validity. The Delphi survey was carried out between February 2024 and April 2024. Panellists were asked to complete each survey round within 2 weeks. Up to two reminders were sent to panellists who did not complete the survey within the 2 weeks. The survey rounds were closed 4 weeks after the initial invitation.

In both Delphi rounds, panellists were requested to indicate their level of agreement or disagreement with the following statement: “*This evaluation indicator should be recommended for inclusion in impact evaluations of all Frontline*,* Intermediate and Advanced FETPs*”. A 5-point Likert rating scale was used to assess the level of agreement, ranging from 1 “Strongly Disagree” to 5 “Strongly Agree” (Strongly Disagree (SD), Disagree (D), Neither Agree nor Disagree (NAD), Agree (A), Strongly Agree (SA)) [29, 30]. Participants were also given the option to select ‘not applicable/unsure’ and were invited to comment on any specific indicator and provide suggestions for additional indicators. If a new indicator was added or substantially modified, it was included in the next round of the Delphi process. Consistent with previous studies, indicators reaching consensus for inclusion or exclusion in the first survey round were removed from the second round [25, 27, 31–35]. During the second round, panellists were shown the first-round results (the proportion of panellists selecting SA, A, NAD, D, and SD for each indicator) and reminded of how they personally had voted during the first round. This approach allowed panellists to re-evaluate the indicators, having the option to change or maintain their original answers. Consensus was determined ‘a priori’ using the median and interquartile range. The median and interquartile range are frequently used measures in Delphi studies and are generally accepted as an objective and rigorous way of determining consensus [20, 34, 35]. The consensus level required for an indicator to be included was defined as a median of 4 or 5 with a lower quartile value of ≥ 4. If an indicator had an upper quartile value of ≤ 2, this indicated there was general disagreement that the indicator should be included by panel members, resulting in the indicator being rejected [34, 35]. 

### Expert review

A final expert review meeting was held via Zoom to discuss the 24 indicators that were neither accepted nor rejected by consensus during the Delphi survey rounds. All panellists who had completed at least one round of the Delphi survey were invited to participate. Before the meeting, panellists were sent the indicators requiring a decision. During the meeting, an anonymous poll was taken, followed by a discussion. The poll was used to gauge the level of consensus for the remaining 24 indicators and focus discussions on indicators without a clear consensus.

## Results

### Stage 1. developing a suite of FETP evaluation indicators

The first stage resulted in the identification of 140 evaluation indicators covering a range of outputs, outcomes and impacts. The indicators were grouped under the 4 levels of change within the evaluation framework; trainees (indicators pertaining to trainees/fellows/residents while undergoing training); graduates (indicators pertaining to graduates or alumni of a FETP); public health system (indicators pertaining to the effect the FETP training on the public health system); and community/general public (indicators pertaining to the effect of the FETP training on the population/community).

### Stage 2. consensus FETP expert panel to review, refine and select core FETP evaluation indicators

#### Panel participation

A total of 36 experts expressed interest or were referred by colleagues to be Delphi panellists; 4 were screened out due to over-representation from the country they represented, resulting in invitations being sent to 32 potential panellists.

There was representation from a range of roles and regions (Tables 1 and 2). Of the 32 invited, 23 (72%) panellists participated in round 1 and 21 (66%) in round 2 (Table 1). There were 7 panellists working at a global level supporting FETP programs in multiple countries around the world. There were 25 panellists representing countries from 5 WHO regions (Table 2).

**Table 1:** Expert panel response rate by expert role and Delphi round, February– April 2024

Experts Role	Number of experts invited	Number (%) participating in Delphi	
		Round 1	Round 2
FETP Director	9	7 (78%)	6 (67%)
Technical Expert	9	4 (44%)	4 (44%)
FETP Evaluation Expert	3	3 (100%)	2 (67%)
Senior Advisor	3	3 (100%)	3 (100%)
TEPHINET Director	2	1 (50%)	1 (50%)
FETP Course Coordinator	2	1 (50%)	1 (50%)
FETP Program Manager	2	2 (100%)	2 (100%)
Regional Director	2	2 (100%)	2 (100%)
**Total**	**32**	**23 (72%)**	**21 (66%)**

**Table 2:** Expert panel participation by region and Delphi round, February – April 2024

Region/Country Represented	Invited	Round 1	Round 2
Global	7	7	5
Eastern Mediterranean Region	4	4	4
South-East Asian Region	11	6	6
African Region	1	0	0
Western Pacific Region	5	2	2
Region of the Americas	4	4	4
**Total**	**32**	**23**	**21**

#### Delphi rounds

**Fig. 2:**
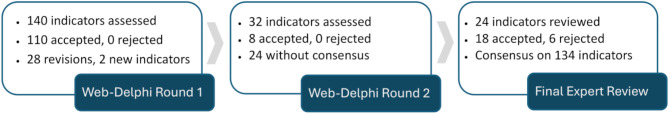
Overview of the modified Delphi process to identify and select evaluation indicators for FETPs

A summary of the two online questionnaire rounds and the final expert review is provided in Fig. 2. At the end of round 1, the panel reached a consensus to include 110 of the 140 indicators (79%) (Table 3). These indicators were included in the final list of recommended indicators and removed from the next Delphi round. During the first round, there were suggestions to clarify the wording (minor modifications) for 28 indicators. For another 18 indicators, suggestions and comments from the panellist resulted in the inclusion of a footnote in the evaluation framework to provide additional context or explanation. Panellists also identified indicators that should be included even though they may not be relevant for all FETPs. These indicators were identified in the evaluation framework with an asterisk and a comment that the indicator may not be relevant or expected for some FETPs or their graduates. Reviewers suggested the addition of two new indicators during round 1, which were included for review in round 2.

At the end of round 2, the panel had reached a consensus on 8 of the 32 indicators (25%). After the two rounds, there was a consensus to accept 118 indicators; none were rejected. The median score attributed to each indicator, along with the interquartile range, is included in Additional file 2, and the comments from reviewers are in Additional file 3. The 24 indicators that did not reach consensus after the two Delphi rounds were taken to the expert panel for final review (Table 3).

Panellists were also asked how often they thought FETPs should conduct an impact evaluation. The majority (*n* = 14, 58%) of panellists suggested conducting an impact evaluation every 5 years, with some panellists suggesting every 2 years (*n* = 4, 17%), every 10 years (*n* = 3, 13%) or every 3 years (*n* = 1, 4%). A small number of panellists recommended that an impact evaluation should follow a significant change in the program structure (*n* = 3, 15%) or governance (*n* = 1, 4%).

**Table 3:** Number (%) of FETP evaluation indicators in each subcategory of the impact evaluation framework reviewed and accepted during the two rounds of the modified Delphi

Level of change		Round 1		Round 2		After 2 Delphi Rounds		
		No. of Indicators	Accepted *n* (%)	No. of Indicators	Accepted *n* (%)	Accepted	Rejected	No consensus
Outputs	Trainees	30	21 (70%)	10^a^	0 (0%)	21	0	10
	Graduates	21	11 (52%)	11^b^	2 (18%)	13	0	9
	Public Health System	21	17 (81%)	4	3 (75%)	20	0	1
	Community / General public	2	2 (100%)	0	-	2	0	0
Outcomes	Trainees	9	9 (100%)	0	-	9	0	0
	Graduates	21	18 (86%)	3	1 (33%)	19	0	2
	Public Health System	19	19 (100%)	0	-	19	0	0
	Community / General public	5	1 (20%)	4	2 (50%)	3	0	2
Impact	Public Health System	9	9 (100%)	0	-	9	0	0
	Community / General public	3	3 (100%)	0	-	3	0	0
**Total**		**140**	**110 (79%)**	**32**	**8 (25%)**	**118**	**0**	**24**

^a^Includes one new indicator from round 1

^b^Includes one new indicator from round 1

#### Final expert review

After the two Delphi survey rounds, a virtual expert review meeting was held with eight Delphi panel experts to discuss the 24 indicators lacking consensus. An initial Zoom poll followed by a discussion resulted in the panel accepting 18 and rejecting six indicators. Additional feedback was provided during the meeting, resulting in wording clarifications for four indicators, merging two similar indicators, and removing one duplicate indicator. The panel also distinguished between higher and lower priority indicators; these were noted in the final impact evaluation framework. The results from the expert meeting were summarised and distributed to the group for final review and comment. Following the two survey rounds and the virtual meeting, a total of 134 indicators made it into the final evaluation framework, with the indicators populating each of the change areas within the impact evaluation framework, grouped by outputs, outcomes and impacts (Additional file 4).

## Discussion

FETPs are central to developing and maintaining the health security workforce at the national, regional and global levels. The ability of countries to detect and respond to emerging public health threats is a requirement under the International Health Regulations, with specific targets for one field epidemiologist per 200,000 population [36]. While the importance of FETPs in strengthening the capability to detect, investigate and respond to public health threats has been widely acknowledged, there is little empirical evidence demonstrating impact. It is imperative that programs develop strong monitoring and evaluation frameworks to demonstrate this impact. The FETP impact evaluation framework and the indicators developed through this consensus process provide an important tool to support program evaluators in assessing impact. This study refined, prioritised and obtained consensus on 134 evaluation indictors for Frontline, Intermediate and Advanced FETPs. It is not intended that all indicators are used in all evaluations. Rather, priority indicators should be identified and selected based on the key evaluation questions developed for each specific FETP evaluation. To our knowledge, this is the first impact evaluation framework and catalogue of evaluation indicators published for FETPs.

The tendency to focus on measuring outputs is a shared experience across the development sector, as they are relatively easy to measure [37]. However, capturing the outcomes and impacts of FETPs is essential to provide the evidence necessary to strengthen, replicate and scale programs. In addition, program donors and beneficiaries are increasingly expecting programs to demonstrate impact and value for money [18]. As the key driver behind FETPs is to improve the health of populations by strengthening the capability to detect, investigate and respond to public health threats, understanding how FETPs contribute to these outcomes is essential. The use of a common framework and set of indicators will bring a level of consistency that has not previously been possible. The evaluation data generated will provide an opportunity to compare and contrast different FETP training models, methods and curricula in order to optimise programs for efficiency and impact. This is especially important in the wake of the COVID-19 pandemic, with calls for a massive scaling up of FETPs to improve the global health architecture in preparation for the next pandemic [38]. 

The modified Delphi process provided a pragmatic and effective method of identifying, refining and selecting evaluation indicators, drawing on the experience of experts in the field. This method assures anonymity throughout the survey rounds, allowing consensus to be sought without prejudice or interpersonal relationships introducing bias [39]. The Delphi technique is a well-established approach for obtaining of a consensus view across subject experts [40]. The whole process was conducted virtually, allowing for easy participation from all regions of the world. The panellists participating represented a range of viewpoints based on their experience within the FETP community at global, regional or national levels. There was a high level of consensus to accept most proposed indicators after the first round, with 110 (79%) accepted based on a pre-determined level of consensus. Although there were suggestions to modify some indicators and merge others, no indicators were rejected during the Delphi survey rounds. This may have been partly due to the rigorous process used to create the initial list of indicators. The involvement of impact evaluation experts and FETP professionals in developing, refining and pre-screening the evaluation indicators eliminated the low-value indicators before they made it into the Delphi process.

While it is good practice to monitor and evaluate every cohort, a full impact evaluation will require far more resources than routine monitoring and evaluation activities. The Better Evaluation organisation states that an impact evaluation should only be undertaken when its intended use can be clearly identified, and there are adequate resources to undertake a sufficiently comprehensive and rigorous impact evaluation [41]. We concur with the majority of the panellists in recommending that FETPs undertake impact evaluations every 5 years. This is a reasonable timeframe to accumulate a sufficiently large sample of graduates, while being soon enough to reliably assess the impact of changes implemented since the previous impact evaluation.

This study has several limitations. Initial recruitment for the Delphi occurred during the Bi-regional TEPHINET Scientific Conference, a conference focused on FETP communities from the South-East Asian and Western Pacific regions. This initial recruitment was biased towards those attending the conference. To counter this initial selection bias, we expanded our invitations to panellists recommended by leaders from within the global FETP community. Although the expert panel was chosen to represent a range of programs from all regions of the world, the sample size was limited to volunteers available during the study timeframe. In the end, there were no panellists representing individual FETPs from the African and European regions. While all the panellists were invited to the final expert review, only eight panellists participated. The results reached in the final round may be biased in favour of those experts who attended the meeting. This bias was reduced to some extent by summarising the results of the virtual meeting and distributing them to the entire group of 32 panellists for final comments and input. Our final virtual meeting removed the element of anonymity, meaning that some dominant individuals could potentially influence the views of others [39, 42, 43]. The absence of a virtual meeting, however, would have limited the opportunity for experts to exchange information and seek clarification in order to generate the best decisions [42, 43]. Consensus methods have other methodological limitations, such as the pressure participants may feel to conform to the group view [44]. 

Despite these limitations, the robust methodology used throughout the development of the impact evaluation framework and evaluation indicators meant that at various stages of the process, there was input from a broad range of stakeholders, resulting in a comprehensive list of indicators that is likely representative of program needs. An earlier (pre-Delphi) version of the evaluation framework and indicators was used to guide the impact evaluations of Frontline, Intermediate and Advanced FETPs in a low-income setting (Papua New Guinea) and a high-income setting (Canada), demonstrating the versatility of this framework.

## Conclusion

This study finalised an impact evaluation framework by obtaining consensus on a comprehensive set of FETP evaluation indicators. These output, outcome, and impact indicators are categorised into thematic areas that follow a high-level FETP theory of change: trainees, graduates, the public health system, and the community. FETP impact evaluations are essential to drive the continuous improvement of FETPs and provide a strong evidence base for scaling and replicating successful training models to meet the evolving demands of global health challenges. The FETP impact evaluation framework and evaluation indicators provide an important tool for guiding and promoting evaluations that move beyond measuring outputs to describing outcomes and impact. The data generated by these impact evaluations will contribute evidence to inform the development of FETP programs to optimally train and equip field epidemiologists around the world.

## Electronic supplementary material

Below is the link to the electronic supplementary material.

Supplementary Material 1

## Data Availability

Data is provided within the manuscript or supplementary information files.

## Abbreviations

FETP: Field epidemiology training program
FELTP: Field epidemiology and laboratory training programs
FETPV: Epidemiology training programs for veterinarians
TEPHINET: Training programs in epidemiology and public health interventions network
SD: Strongly disagree
D: Disagree
NAD: Neither agree nor disagree
A: Agree
