# Supplementary material for: Priority indicators for evaluating the impact of field epidemiology training programs – results of a global modified Delphi study
Source: BMC Public Health. 2025 Feb 17;25:635. doi: 10.1186/s12889-025-21816-2 (PMC11831827; doi:10.1186/s12889-025-21816-2)
Supplement: Supplementary file 1 — Supplementary Material 1 [file 12889_2025_21816_MOESM1_ESM.docx]

**Additional file 1.** Questionnaire use to develop the online web-Delhi using Welphi online software

**Identifying core indicators for Field Epidemiology Training Program impact evaluation**

**CONSENT**

The University of Newcastle are conducting a study to identify a core set of indictors for Field Epidemiology Training Program evaluation.

You will have been sent an information sheet outlining the study, as well as a consent form. Have you read the information sheet and provided your consent to participate in this Delphi Study?

- Yes
- No

**INTRODUCTION**

Please provide your information below. This information will only be used to identify participants for round 2 of the survey. No identifiable responses will be shared with any other participants or appear in any report.

1. Name:
2. Email address:

**OVERVIEW OF QUESTIONNAIRE**

The purpose of this questionnaire is to gain consensus on a minimum set of core (priority) FETP evaluation indicators that can be recommended for inclusion in FETP impact evaluations. Having a set of recommended indicators will provide guidance to programs embarking on evaluation activities and support a higher degree of inter-program comparison.

An initial list of indictors was generated through background research, literature reviews, FETP theories of change, expert review and input from FETP faculty representing several programs. This quite extensive list of evaluation indictors covers outputs, outcomes and impacts.

From your perspective, please assess the value of including each of the listed indicators as core indicators for inclusion in evaluations of Frontline, Intermediate or Advanced FETPs.

It is understood that FETPs will almost always include additional evaluation indicators that are specific to their program. Your task is to select priority indicators which you think should be always be included when evaluating Frontline, Intermediate or Advanced FETPs. Specialised FETPs (e.g. FETPs focused on One Health, non-communicable disease, laboratory) fall outside the scope of this Delphi.

The outcome of this study will be to publish a list of recommended indicators for FETP impact evaluations.

**SELECTION OF CORE INDICATORS**

The indicators are listed by the following three categories:

1. Outputs - Products, projects, or activities which result from the training program
2. Outcomes - Short-term and medium-term effects of the training program
3. Impacts - Longer term effects produced by the training program

Under each category, the indicators have been aligned to one of four groups:

1. Fellows – indicators relating to fellows/trainees/residents while undergoing training
2. Graduates – indicators relating to graduates or alumni of FETP
3. Public Health System – indicators relating to the effect the FETP training on the public health system
4. Community – indicators relating to the effect of the FETP training on the population/community

You may also add additional indicators to the relevant category/group. These additional indictors will be reviewed and included in the next questionnaire round.

Please use the comments box for any comments relating to specific indicators.

For each of the FETP evaluation indicators, you are responding to the following statement: *This evaluation indicator should be recommended for inclusion in impact evaluations of all Frontline, Intermediate and Advanced FETPs*

Your response will be 'Strongly Agree', 'Agree', 'Neither Agree nor Disagree', 'Disagree', 'Strongly Disagree' or 'Don't know/Don't want to answer'.

This first round will take approximately 45-60 minutes to complete. There are 3 main pages listing indicators by outputs, outcomes and impact. You do not have to complete the online questionnaire in one sitting. If you partially complete a page and close your browser, your responses will be saved. You can use the same link to re-access your questionnaire.

We really appreciate your time and expertise in completing this Delphi questionnaire. Thank you.

Please complete the online questionnaire round by Wednesday Feb 21, 2024.

Please press "Next" to start.

**This evaluation indicator should be recommended for inclusion in impact evaluations of all Frontline, Intermediate and Advanced Field Epidemiology Training programs FETPs:**

| **OUTPUTS** | | Strongly Disagree | Disagree | Neither Agree or Disagree | Agree | Strongly Agree | Comments |
| --- | --- | --- | --- | --- | --- | --- | --- |
| **FELLOWS** | **Training Participation** | | | | | |  |
|  | Gender breakdown of fellows |  |  |  |  |  |  |
|  | Number/percentage of fellows enrolled in training by role, position, and workplace location |  |  |  |  |  |  |
|  | Number/percentage of fellows linked with public health mentors/supervisors |  |  |  |  |  |  |
|  | **Training Quality and Relevance** | | | | | |  |
|  | Number of hours of training in a classroom type setting (including virtual training) |  |  |  |  |  |  |
|  | Total length of time in the field/workplace (in weeks) |  |  |  |  |  |  |
|  | Number/percentage of fellows reporting enjoyable learning experience |  |  |  |  |  |  |
|  | Number/percentage of fellows reporting training relevance to current role |  |  |  |  |  |  |
|  | Number/percentage of fellows reporting improved field knowledge and skills in key field epidemiology competencies |  |  |  |  |  |  |
|  | Number/percentage of fellows that meet program’s core competencies |  |  |  |  |  |  |
|  | Approximate number of hours of direct one-on-one mentoring provided to fellows |  |  |  |  |  |  |
|  | **Operational Research, Surveillance, Outbreak Investigation** |  |  |  |  |  |  |
|  | Number of operational research studies completed by fellows /  Number/percentage of fellows completing operational research studies |  |  |  |  |  |  |
|  | Number of surveillance systems evaluated by fellows /  Number/percentage of fellows evaluating surveillance systems |  |  |  |  |  |  |
|  | Number of outbreak investigations led by fellows /  Number/percentage of fellows leading outbreak investigations |  |  |  |  |  |  |
|  | Number of outbreaks investigations supported by fellows /  Number/percentage of fellows supporting outbreak investigations |  |  |  |  |  |  |
|  | Number of national mobilizations involving fellows /  Number/percentage of fellows mobilized nationally |  |  |  |  |  |  |
|  | Number of international mobilizations involving fellows /  Number/percentage of fellows mobilized internationally |  |  |  |  |  |  |
|  | Number/percentage of deployed fellows who undertake post-deployment debriefs |  |  |  |  |  |  |
|  | Number/percentage of fellow mobilizations meeting needs of requestor |  |  |  |  |  |  |
|  | **Written Products, Presentations and Processes** | | | | | |  |
|  | Number of epidemiological reports prepared by fellows /  Number/percentage of fellows preparing epidemiological reports |  |  |  |  |  |  |
|  | Number of policy briefs written by fellows /  Number/percentage of fellows writing policy briefs |  |  |  |  |  |  |
|  | Number of policies updated or developed by fellows /  Number/percentage of fellows updating or developing policies |  |  |  |  |  |  |
|  | Number of health program recommendations made by fellows /  Number/percentage of fellows making health program recommendations |  |  |  |  |  |  |
|  | Number of papers published with fellows as lead authors /  Number/percentage of fellows publishing papers as lead authors |  |  |  |  |  |  |
|  | Number of papers published with fellows as co-authors /  Number/percentage of fellows publishing papers as co-authors |  |  |  |  |  |  |
|  | Number of presentations within fellows’ workplace or placement site /  Number/percentage of fellows giving presentations in workplace / placement site |  |  |  |  |  |  |
|  | Number of presentations outside of fellows’ workplace/placement site /  Number/percentage of fellows giving presentation outside workplace / placement site |  |  |  |  |  |  |
|  | Number of conference abstracts submitted by fellows being accepted /  Number/percentage of fellows having conference abstracts accepted |  |  |  |  |  |  |
|  | Number of conference presentations given by fellows /  Number/percentage of fellows giving conference presentations |  |  |  |  |  |  |
|  | Number of work-related processes or procedures updated or developed by fellows / Number/percentage of fellows updating or developing work-related processes or procedures |  |  |  |  |  |  |
|  | ADDITIONAL INDICATOR, please specify:  (note: multiple additional indictors permitted) |  |  |  |  |  |  |
| **GRADUATES** | **Graduates** | | | | | |  |
|  | Gender breakdown of graduates |  |  |  |  |  |  |
|  | Number/percentage of graduates completing FETP training by role, position and workplace location |  |  |  |  |  |  |
|  | Number/percentage of graduates undertaking continuing education / professional development training |  |  |  |  |  |  |
|  | Number/percentage of graduates completing higher level training (course name and level, e.g. leadership training, Master of Public Health, PhD, etc) |  |  |  |  |  |  |
|  | Number/percentage of graduates given additional responsibilities in their employment because of their field epidemiology training (describe additional responsibilities) |  |  |  |  |  |  |
|  | Number/percentage of graduates receiving a promotion within 12 months of graduating |  |  |  |  |  |  |
|  | **Operational Research, Surveillance, Outbreak Investigation** (for each indicator, record length of time since graduation to enable yearly average to be calculated) | | | | | | |
|  | Number of operational research studies completed by graduates /  Number/percentage of graduates completing operational research studies |  |  |  |  |  |  |
|  | Number of surveillance systems evaluated by graduates /  Number/percentage of graduates evaluating surveillance systems |  |  |  |  |  |  |
|  | Number of outbreak investigations led by graduates /  Number/percentage of graduates leading outbreak investigations |  |  |  |  |  |  |
|  | Number of outbreaks investigations supported by graduates /  Number/percentage of graduates supporting outbreak investigations |  |  |  |  |  |  |
|  | Number of national mobilizations involving graduates  Number/percentage of graduates mobilized nationally |  |  |  |  |  |  |
|  | Number of international mobilizations involving graduates  Number/percentage of graduates mobilized internationally |  |  |  |  |  |  |
|  | Number/percentage of deployed graduates who undertake post-deployment debriefs |  |  |  |  |  |  |
|  | Number/percentage of graduate mobilizations that meet the needs of requestor |  |  |  |  |  |  |
|  | Number/percentage of graduates that feel confident to respond to emerging issues in public health |  |  |  |  |  |  |
|  | Number/percentage of graduates supporting an Incident Management System (IMS) during a public health emergency response |  |  |  |  |  |  |
|  | **Written Products, Presentations and Processes** | | | | | |  |
|  | Number of epidemiological reports prepared by graduates /  Number/percentage of graduates preparing epidemiological reports |  |  |  |  |  |  |
|  | Number of policy briefs written by graduates /  Number/percentage of graduates writing policy briefs |  |  |  |  |  |  |
|  | Number of policies updated or developed by graduates /  Number/percentage of graduates updating or developing policies |  |  |  |  |  |  |
|  | Number of health program recommendations made by graduates /  Number/percentage of graduates making health program recommendations |  |  |  |  |  |  |
|  | Number of papers published with graduates as lead authors /  Number/percentage of graduates publishing papers as lead authors |  |  |  |  |  |  |
|  | Number of papers published with graduates as co-authors /  Number/percentage of graduates publishing papers as co-authors |  |  |  |  |  |  |
|  | Number of presentations within graduates’ workplace or placement site /  Number/percentage of graduates giving presentations in workplace or placement site |  |  |  |  |  |  |
|  | Number of presentations outside of graduates’ workplace or placement site /  Number/percentage of graduates giving presentation outside workplace or placement site |  |  |  |  |  |  |
|  | Number of conference abstracts submitted by graduates being accepted /  Number/percentage of graduates having conference abstracts accepted |  |  |  |  |  |  |
|  | Number of conference presentations given by graduates /  Number/percentage of graduates giving conference presentations |  |  |  |  |  |  |
|  | Number of work-related processes or procedures updated or developed by graduates / Number/percentage of graduates updating or developing work-related processes or procedures |  |  |  |  |  |  |
|  | ADDITIONAL INDICATOR, please specify:  (note: multiple additional indictors permitted) |  |  |  |  |  |  |
| **PUBLIC HEALTH SYSTEM** | **Field Epidemiology Workforce** | | | | | |  |
|  | Number/percentage of graduates placed across different tiers of health system (e.g. district, provincial, national levels) |  |  |  |  |  |  |
|  | Number/percentage of [districts / provinces] in country with graduates |  |  |  |  |  |  |
|  | Number/percentage of graduates employed by a government institution |  |  |  |  |  |  |
|  | Number/percentage of graduates employed by a non-governmental organization |  |  |  |  |  |  |
|  | Number/percentage of graduates employed by an academic institution |  |  |  |  |  |  |
|  | Number/percentage of graduates employed in epidemiology or applied public health related positions |  |  |  |  |  |  |
|  | Number/percentage of graduates who are members of national, regional or international public health committees or working groups |  |  |  |  |  |  |
|  | **Support for Surveillance and Outbreak Response** | | | | | |  |
|  | Number of disease surveillance systems strengthened by fellows and graduates |  |  |  |  |  |  |
|  | Number of outbreak response systems and practices strengthened by fellows and graduates |  |  |  |  |  |  |
|  | Number/percentage of outbreaks detected from disease surveillance systems |  |  |  |  |  |  |
|  | Number/percentage of communicable disease outbreaks where an investigation commenced within 24, 48 and 72 hours |  |  |  |  |  |  |
|  | **Support for Health Systems** | | | | | |  |
|  | Number of evidence-based recommendations implemented by fellows and graduates /  Number/percentage of fellows and graduates implementing evidence-based recommendations |  |  |  |  |  |  |
|  | Number of policy recommendations implemented by fellows and graduates /  Number/percentage of fellows and graduates implementing policy recommendations |  |  |  |  |  |  |
|  | Number of health program recommendations implemented by fellows and graduates /  Number/percentage of fellows and graduates implementing health program recommendations |  |  |  |  |  |  |
|  | Number of work-related processes or procedures implemented by fellows and graduates / Number/percentage of fellows and graduates implementing work-related processes or procedures |  |  |  |  |  |  |
|  | **FETP Support** | | | | | |  |
|  | Number/percentage of FETP trainers and mentors who are graduates of the program |  |  |  |  |  |  |
|  | Number/percentage of FETP staff (e.g Director, convenor) who are graduates of the program |  |  |  |  |  |  |
|  | FETP is recognised in official governmental/intuitional planning documents |  |  |  |  |  |  |
|  | FETP steering committee is established and functional |  |  |  |  |  |  |
|  | Number/percentage of FETP positions fully staffed |  |  |  |  |  |  |
|  | Percentage of FETP activities funded by national government |  |  |  |  |  |  |
|  | FETP accredited by TEPHINET |  |  |  |  |  |  |
|  | ADDITIONAL INDICATOR, please specify:  (note: multiple additional indictors permitted) |  |  |  |  |  |  |
| **COMMUNITY** | **Community Level Outputs** |  |  |  |  |  |  |
|  | Number of new or strengthened community based public health programs conducted or coordinated by fellows or graduates |  |  |  |  |  |  |
|  | Number of community engagement activities conducted by fellows or graduates |  |  |  |  |  |  |
|  | Number of community awareness / training activities conducted by fellows or graduates |  |  |  |  |  |  |
|  | Number of public health events notified to by the community |  |  |  |  |  |  |
|  | ADDITIONAL INDICATOR, please specify:  (note: multiple additional indictors permitted) |  |  |  |  |  |  |

| **OUTCOMES** | | Strongly Disagree | Disagree | Neither Agree or Disagree | Agree | Strongly Agree |  |
| --- | --- | --- | --- | --- | --- | --- | --- |
| **FELLOWS** | **Trained Field Epidemiologists**​ | | | | | |  |
|  | Fellows demonstrate the application of field epi competencies throughout the training |  |  |  |  |  |  |
|  | Fellows are provided quality mentoring throughout training program |  |  |  |  |  |  |
|  | Fellows are confident in applying their knowledge and skills in their workplace / placement site |  |  |  |  |  |  |
|  | **Competent Field Epidemiologists** | | | | | |  |
|  | Fellows undertake field projects, such as surveillance evaluations, operational research, or interventions |  |  |  |  |  |  |
|  | Fellows contribute to improvements in work-related processes or procedures at their workplace/placements site |  |  |  |  |  |  |
|  | Fellows improve a disease surveillance system(s) |  |  |  |  |  |  |
|  | Fellows analyse and interpret surveillance data to inform decision making |  |  |  |  |  |  |
|  | Fellows investigate outbreaks by appropriately following the steps of an outbreak investigation |  |  |  |  |  |  |
|  | Fellows implement or support the implementation of appropriate control measures during outbreak investigations |  |  |  |  |  |  |
|  | Fellows report unintended positive and/or negative consequences of FETP |  |  |  |  |  |  |
|  | ADDITIONAL INDICATOR, please specify:  (note: multiple additional indictors permitted) |  |  |  |  |  |  |
| **GRADUATES** | **Skilled Field Epi workforce**​ | | | | | |  |
|  | Graduates employed in positions where field epi knowledge and skills are required |  |  |  |  |  |  |
|  | Graduates provided opportunities to apply skills and knowledge in the workplace |  |  |  |  |  |  |
|  | Graduates confident in applying knowledge and skills in the workplace |  |  |  |  |  |  |
|  | Graduates improve the overall quality of their work |  |  |  |  |  |  |
|  | Graduates progress in their careers as a result of their graduation from a FETP |  |  |  |  |  |  |
|  | **Contributing Field Epi graduates**​ | | | | | |  |
|  | Graduates undertake field projects, such as surveillance evaluations, operational research, or interventions |  |  |  |  |  |  |
|  | Graduates improve disease surveillance systems |  |  |  |  |  |  |
|  | Graduates routinely analyse and interpret surveillance data to inform decision making |  |  |  |  |  |  |
|  | Graduates investigate outbreaks by appropriately following the steps of an outbreak investigation |  |  |  |  |  |  |
|  | Graduates implement or support the implementation of appropriate control measures during outbreak investigations |  |  |  |  |  |  |
|  | Graduates use evidence-based decision-making processes in the workplace |  |  |  |  |  |  |
|  | Graduates are developing or contributing to the development of public health policy |  |  |  |  |  |  |
|  | Graduates are introducing improved ways of delivering public health programs |  |  |  |  |  |  |
|  | Graduates contribute to improvements in work-related processes or procedures at their workplace/placements site |  |  |  |  |  |  |
|  | Graduates are recognized, utilized and have influence in the workplace |  |  |  |  |  |  |
|  | Graduates transfer field epidemiology knowledge and skills to others through training and mentoring |  |  |  |  |  |  |
|  | Graduates applied field epidemiology knowledge and skills to other fields |  |  |  |  |  |  |
|  | Graduates contribute to raising the profile of field epidemiology, surveillance and/or public health |  |  |  |  |  |  |
|  | Graduates report unintended positive and/or negative consequences of FETP |  |  |  |  |  |  |
|  | **Networks and Partnerships** | | | | | |  |
|  | Graduates actively engage with and contribute to an FETP alumni network |  |  |  |  |  |  |
|  | Graduates develop and engage in networks and partnership to improve public health practice |  |  |  |  |  |  |
|  | Graduates share field epidemiology learnings and good practices with public health colleagues |  |  |  |  |  |  |
|  | ADDITIONAL INDICATOR, please specify:  (note: multiple additional indictors permitted) |  |  |  |  |  |  |
| **PUBLIC HEALTH SYSTEM** | **Influential Field Epi workforce** | | | | | |  |
|  | Established career pathway for graduates |  |  |  |  |  |  |
|  | Graduates are in public health leadership roles in governmental departments and non-governmental organizations |  |  |  |  |  |  |
|  | Key surveillance and disease control positions at all tiers of government are occupied by FETP graduates |  |  |  |  |  |  |
|  | Skills of graduates are maintained and continually applied |  |  |  |  |  |  |
|  | Decision makers confident in and engaged with FETP graduates and the services they provide |  |  |  |  |  |  |
|  | Graduates develop and deliver field epidemiology related training activities to workplace colleagues |  |  |  |  |  |  |
|  | Graduates are public health influencers in their workplace and the communities they serve |  |  |  |  |  |  |
|  | **Health Systems Strengthened** | | | | | |  |
|  | Graduates routinely conduct field projects/operational research to understand and address key public health challenges |  |  |  |  |  |  |
|  | Graduates routinely design and implement public health interventions to address public health challenges |  |  |  |  |  |  |
|  | Graduates effectively engage with communities when planning and delivering public health programs |  |  |  |  |  |  |
|  | Decision makers utilize the evidence generated by graduates to improve public health programming |  |  |  |  |  |  |
|  | Graduates are routinely using evidence-based decision making to inform guidelines, policy & programmatic activities |  |  |  |  |  |  |
|  | Graduates are driving innovation and service improvements |  |  |  |  |  |  |
|  | **Stronger Surveillance and Outbreak Response** | | | | | |  |
|  | Graduates contribute to improved surveillance resulting in improved public health programming |  |  |  |  |  |  |
|  | Graduates contribute to improved surveillance resulting in improved outbreak detection and response |  |  |  |  |  |  |
|  | Graduates provide a response-ready workforce for outbreak and public health emergency response activities nationally and/or internationally |  |  |  |  |  |  |
|  | Graduates effectively engage with communities when investigating outbreaks and responding to public health threats |  |  |  |  |  |  |
|  | **Graduate Support for FETP(s)** | | | | | |  |
|  | Established pathway for FETP graduates to become FETP trainers, mentors, and program staff |  |  |  |  |  |  |
|  | Graduates contribute to the national FETP(s) as trainers, mentors, and program staff |  |  |  |  |  |  |
|  | ADDITIONAL INDICATOR, please specify:  (note: multiple additional indictors permitted) |  |  |  |  |  |  |
| **COMMUNITY** | **Community Level Outcomes** |  |  |  |  |  |  |
|  | Graduates contribute to improved access to public health services |  |  |  |  |  |  |
|  | Graduates contribute to improved quality of public health services |  |  |  |  |  |  |
|  | Graduates contribute to improve health program key performance indicators (KPIs), such as vaccine coverage, supervised deliveries, etc |  |  |  |  |  |  |
|  | Graduates contribute to improved community engagement resulting in community involvement in public health decision making process affecting them |  |  |  |  |  |  |
|  | Graduates continued to improved health literacy within community |  |  |  |  |  |  |
|  | ADDITIONAL INDICATOR, please specify:  (note: multiple additional indictors permitted) |  |  |  |  |  |  |

| **IMPACTS** | | Strongly Disagree | Disagree | Neither Agree or Disagree | Agree | Strongly Agree |  |
| --- | --- | --- | --- | --- | --- | --- | --- |
| **PUBLIC HEALTH SYSTEM** | **Strong Health Systems** | | | | | |  |
|  | FETP faculty and graduates contribute to the generation of public health intelligence and evidence-based recommendations to improve public health |  |  |  |  |  |  |
|  | FETP faculty and graduates contribute to an evidence-based decision-making culture that drives public health programming and practice |  |  |  |  |  |  |
|  | FETP faculty and graduates contribute to the development of evidence based public health policies and practices that are accepted, resourced, and implemented |  |  |  |  |  |  |
|  | FETP faculty and graduates contribute to strong disease surveillance systems that guide public health programming and consistently supports the early detection & response to public health threats |  |  |  |  |  |  |
|  | FETP faculty and graduates are influential in advocating for political and financial support for public health at all levels of the health system |  |  |  |  |  |  |
|  | FETP faculty and graduates contribute to a culture of respectful community engagement when designing and delivering public health programs and response activities |  |  |  |  |  |  |
|  | **Sustainable FETP(s)** | | | | | |  |
|  | FETP is institutionalized, adequately funded and nationally recognised as an important public health workforce development program |  |  |  |  |  |  |
|  | FETP run & delivered by national staff without the need for external support |  |  |  |  |  |  |
|  | FETP program is influential across public health networks nationally and internationally |  |  |  |  |  |  |
|  | ADDITIONAL INDICATOR, please specify:  (note: multiple additional indictors permitted) |  |  |  |  |  |  |
| **COMMUNITY** | **Community Level Impacts** |  |  |  |  |  |  |
|  | FETP faculty and graduates contribute to reduced outbreak related morbidity and mortality through timely and effective outbreak response activities |  |  |  |  |  |  |
|  | FETP faculty and graduates contribute to reduced morbidity and mortality through improved access to and provision of health services |  |  |  |  |  |  |
|  | FETP faculty and graduates contribute to reduced morbidity and mortality through improved public health program design and delivery |  |  |  |  |  |  |
|  | ADDITIONAL INDICATOR, please specify:  (note: multiple additional indictors permitted) |  |  |  |  |  |  |

How often do you recommend a FETP conduct an impact evaluation?

 Every 2 years

 Every 5 years

 Every 10 years

 After the FETP implements a significant change

 Other, please specify:

Thanks for completing this round of the Delphi. If you would like to share anything further regarding FETP impact evaluation or this Delphi process, please let us know in the text box below

| **Additional comments / Feedback** |  |
| --- | --- |

Thank you for your time and input, it is greatly appreciated. You will have the opportunity to review and revised your selection and input in the next round of the Delphi process. You will also see the summary results from the other panellist during the second phase. We will in contact via email in approximately 3 weeks.

**Additional file 2.** Median score, upper and lower quartiles for each indicator.

| **No.** | **OUTPUT INDICATORS - ROUND ONE** | **Median** | **Upper** | **Lower** | **Status** |
| --- | --- | --- | --- | --- | --- |
| 1 | Gender breakdown of trainees | 5 | 5 | 4 | Accepted |
| 2 | Number/percentage of trainees enrolled in training by role, position, and workplace location | 5 | 5 | 4.5 | Accepted |
| 3 | Number/percentage of trainees linked with public health mentors/supervisors | 5 | 5 | 4 | Accepted |
| 4 | Number of hours of training in a classroom type setting (including virtual training) | 4 | 5 | 4 | Accepted |
| 5 | Total length of time in the field/workplace (in weeks) | 5 | 5 | 4 | Accepted |
| 6 | Number/percentage of trainees reporting enjoyable learning experience | 3 | 4 | 3 | Undetermined |
| 7 | Number/percentage of trainees reporting training relevance to current role | 4 | 5 | 4 | Accepted |
| 8 | Number/percentage of trainees reporting improved field knowledge and skills in key field epidemiology competencies | 5 | 5 | 4 | Accepted |
| 9 | Number/percentage of trainees that meet program’s core competencies | 5 | 5 | 5 | Accepted |
| 10 | Approximate number of hours of direct one-on-one mentoring provided to trainees | 4 | 4 | 3 | Undetermined |
| 11 | Number of operational research studies completed by trainees [Number/percentage of trainees completing operational research studies] | 4 | 5 | 4 | Accepted |
| 12 | Number of surveillance systems evaluated by trainees [Number/percentage of trainees evaluating surveillance systems] | 4 | 5 | 4 | Accepted |
| 13 | Number of surveillance systems designed and established by trainees | 5 | 5 | 5 | Accepted |
| 14 | Number of outbreak investigations led by trainees [Number/percentage of trainees leading outbreak investigations] | 5 | 5 | 4 | Accepted |
| 15 | Number of outbreaks investigations supported by trainees [Number/percentage of trainees supporting outbreak investigations] | 5 | 5 | 4 | Accepted |
| 16 | Number of national mobilizations involving trainees [Number/percentage of trainees mobilized nationally] | 4 | 5 | 4 | Accepted |
| 17 | Number of international mobilizations involving trainees [Number/percentage of trainees mobilized internationally] | 4 | 5 | 3 | Undetermined |
| 18 | Number/percentage of deployed trainees who undertake post-deployment debriefs | 3.5 | 4 | 3 | Undetermined |
| 19 | Number/percentage of fellow mobilizations meeting needs of person or organization requesting FETP fellow support for a response | 4 | 5 | 4 | Accepted |
| 20 | Number of epidemiological reports prepared by trainees [Number/percentage of trainees preparing epidemiological reports] | 4 | 5 | 4 | Accepted |
| 21 | Number of policy briefs written by trainees [Number/percentage of trainees writing policy briefs] | 4 | 4 | 3 | Undetermined |
| 22 | Number of policies updated or developed by trainees [Number/percentage of trainees updating or developing policies] | 3.5 | 4 | 3 | Undetermined |
| 23 | Number of recommendations made by trainees that were implemented [Number/percentage of trainees making health program recommendations that were implemented] | 4 | 5 | 4 | Accepted |
| 24 | Number of papers published with trainees as lead authors [Number/percentage of trainees publishing papers as lead authors] | 4 | 5 | 3 | Undetermined |
| 25 | Number of papers published with trainees as co-authors [Number/percentage of trainees publishing papers as co-authors] | 4 | 4 | 3 | Undetermined |
| 26 | Number of FETP-related presentations given by fellows within their workplace/placement site [Number/percentage of trainees giving FETP-related presentations within their workplace or placement site] | 4 | 5 | 4 | Accepted |
| 27 | Number of trainees presentations at national, regional, international meetings or conferences [Number/percentage of trainees giving presentations at national, regional, international meetings or conferences] | 4 | 5 | 4 | Accepted |
| 28 | Number of conference abstracts submitted by trainees being accepted [Number/percentage of trainees having conference abstracts accepted] | 4 | 5 | 4 | Accepted |
| 29 | Number of conference presentations given by trainees [Number/percentage of trainees giving conference presentations] | 4 | 5 | 4 | Accepted |
| 30 | Number of work-related processes, policies or guidance documents updated or developed by trainees [Number/percentage of trainees updating or developing work-related processes, policies or guidance documents] | 4 | 4 | 3 | Undetermined |
| 31 | Gender breakdown of graduates | 5 | 5 | 4 | Accepted |
| 32 | Number/percentage of graduates completing FETP training by role, position and workplace location | 4 | 5 | 4 | Accepted |
| 33 | Number/percentage of graduates completing higher level training / professional development (course name and level, e.g. leadership training, Master of Public Health, PhD, etc) | 4 | 4.5 | 3 | Undetermined |
| 34 | Number/percentage of graduates given additional responsibilities in their employment because of their field epidemiology training (describe additional responsibilities) | 5 | 5 | 4 | Accepted |
| 35 | Number/percentage of graduates receiving a promotion within 12 months of graduating | 4 | 4 | 4 | Accepted |
| 36 | Number of operational research studies completed by graduates [Number/percentage of graduates completing operational research studies] | 4 | 4.75 | 3 | Undetermined |
| 37 | Number of surveillance systems evaluated by graduates [Number/percentage of graduates evaluating surveillance systems] | 4 | 5 | 4 | Accepted |
| 38 | Number of outbreak investigations led by graduates [Number/percentage of graduates leading outbreak investigations] | 5 | 5 | 4 | Accepted |
| 39 | Number of outbreaks investigations supported by graduates [Number/percentage of graduates supporting outbreak investigations] | 4.5 | 5 | 4 | Accepted |
| 40 | Number of national mobilizations involving graduates [Number/percentage of graduates mobilized nationally] | 4 | 5 | 4 | Accepted |
| 41 | Number of international mobilizations involving graduates [Number/percentage of graduates mobilized internationally] | 4 | 4.75 | 3 | Undetermined |
| 42 | Number/percentage of deployed graduates who undertake post-deployment debriefs | 3 | 4 | 3 | Undetermined |
| 43 | Number/percentage of graduates supporting an Incident Management System (IMS) during a public health emergency response | 4 | 5 | 4 | Accepted |
| 44 | Number/percentage of graduates reporting that the development of epidemiological reports is part of their current role | 4 | 5 | 4 | Accepted |
| 45 | Number/percentage of graduates reporting that the development of policy briefs is part of their current role | 4 | 5 | 3 | Undetermined |
| 46 | Number/percentage of graduates reporting that improving public health programs is part of their current role | 4 | 5 | 4 | Accepted |
| 47 | Number of papers published with graduates as lead authors [Number/percentage of graduates publishing papers as lead authors] | 4 | 4 | 3.25 | Undetermined |
| 48 | Number of papers published with graduates as co-authors [Number/percentage of graduates publishing papers as co-authors] | 4 | 4 | 3.25 | Undetermined |
| 49 | Number of conference abstracts submitted by graduates being accepted [Number/percentage of graduates having conference abstracts accepted] | 4 | 4 | 3 | Undetermined |
| 50 | Number of conference presentations given by graduates [Number/percentage of graduates giving conference presentations] | 4 | 4.75 | 3.25 | Undetermined |
| 51 | Number/percentage of graduates reporting that updating or developing work-related processes, policies or guidance documents is part of their current role | 4 | 5 | 3 | Undetermined |
| 52 | Number/percentage of graduates working across different tiers of health system (e.g. district, provincial, national levels) | 5 | 5 | 4 | Accepted |
| 53 | Number/percentage of [districts /provinces] in country with graduates | 5 | 5 | 4 | Accepted |
| 54 | Number/percentage of graduates employed by role, position, workplace type and location | 4 | 5 | 4 | Accepted |
| 55 | Number/percentage of graduates who are members of national, regional or international public health committees or working groups | 4 | 5 | 3 | Undetermined |
| 56 | Number of disease surveillance systems strengthened by trainees and graduates | 4 | 5 | 4 | Accepted |
| 57 | Number of outbreak response systems and practices strengthened by trainees and graduates | 4 | 5 | 4 | Accepted |
| 58 | Number of disease surveillance systems strengthened / key performance indicators improved | 4 | 5 | 3 | Undetermined |
| 59 | Number/percentage of graduate workplaces reporting improvements in surveillance | 4 | 4.75 | 4 | Accepted |
| 60 | Number/percentage of graduate workplaces reporting improvements in outbreak response | 4 | 4.75 | 4 | Accepted |
| 61 | Number/percentage of communicable disease outbreaks where an investigation commenced within 24, 48 and 72 hours | 4 | 4 | 3 | Undetermined |
| 62 | Number/percentage of communicable disease outbreaks meeting 7-1-7 targets | 4 | 4 | 3 | Undetermined |
| 63 | Number/percentage of evidence-based recommendations, developed by trainees and graduates, that have been implemented | 4 | 5 | 4 | Accepted |
| 64 | Number/percentage of work-related processes, policies or guidance documents developed by fellow and graduates that have been implemented | 4 | 4 | 4 | Accepted |
| 65 | Number/percentage of placement sites/workplaces reporting improvement in health systems due to work by trainees/graduates (e.g. strengthened surveillance, outbreak response, program delivery, etc) | 5 | 5 | 4 | Accepted |
| 66 | Number/percentage of FETP trainers and mentors who are graduates of the program | 5 | 5 | 4 | Accepted |
| 67 | Number/percentage of FETP staff (e.g. Director, Convenor) who are graduates of the program | 4 | 5 | 4 | Accepted |
| 68 | FETP is recognised in official governmental/institutional planning documents | 5 | 5 | 4.25 | Accepted |
| 69 | FETP steering committee is established and functional | 4 | 5 | 4 | Accepted |
| 70 | Number/percentage of FETP positions fully staffed | 4 | 5 | 4 | Accepted |
| 71 | Percentage of FETP budget funded by the national government | 4 | 5 | 4 | Accepted |
| 72 | FETP accredited by TEPHINET | 4 | 5 | 4 | Accepted |
| 73 | Number of new or strengthened community/population-based public health programs conducted or coordinated by trainees/graduates | 4 | 4 | 4 | Accepted |
| 74 | Number of community/general public engagement activities conducted by trainees or graduates | 4 | 4 | 4 | Accepted |
| **No.** | **OUTCOME INDICATORS - ROUND ONE** | **Median** | **Upper** | **Lower** | **Status** |
| 75 | Trainees demonstrate the application of field epi competencies throughout the training | 5 | 5 | 4 | Accepted |
| 76 | Trainees are confident in applying their knowledge and skills in their workplace/placement site | 5 | 5 | 4 | Accepted |
| 77 | Trainees report unintended positive and/or negative consequences of FETP (e.g. fellow given new work opportunity because skills (positive consequence); fellow works overtime in order to manage both regular work and FETP assignments (negative consequences), etc) | 4 | 5 | 4 | Accepted |
| 78 | Trainees undertake field projects, such as surveillance evaluations, operational research, or interventions | 4.5 | 5 | 4 | Accepted |
| 79 | Trainees contribute to improvements in work-related processes, policies or guidance documents at their workplace/placements site | 4 | 5 | 4 | Accepted |
| 80 | Trainees improve a surveillance system or component of a surveillance system | 5 | 5 | 4 | Accepted |
| 81 | Trainees analyse and interpret surveillance data to inform decision-making | 5 | 5 | 4 | Accepted |
| 82 | Trainees investigate outbreaks by appropriately following the steps of an outbreak investigation | 5 | 5 | 4 | Accepted |
| 83 | Trainees implement or support the implementation of appropriate control measures during outbreak investigations | 4 | 5 | 4 | Accepted |
| 84 | Graduates employed in positions where field epi knowledge and skills are required | 5 | 5 | 4 | Accepted |
| 85 | Graduates provided opportunities to apply skills and knowledge in the workplace | 5 | 5 | 4 | Accepted |
| 86 | Graduates confident in applying knowledge and skills in the workplace | 5 | 5 | 4 | Accepted |
| 87 | Graduates progress in their careers as a result of their graduation from a FETP | 4 | 5 | 4 | Accepted |
| 88 | Graduates undertake field projects, such as surveillance evaluations, operational research, or interventions to improve health systems or health outcomes | 4 | 5 | 4 | Accepted |
| 89 | Graduates improve surveillance systems or components of a surveillance system | 4 | 5 | 4 | Accepted |
| 90 | Graduates routinely analyse and interpret surveillance data to inform decision making | 4 | 5 | 4 | Accepted |
| 91 | Graduates investigate outbreaks by appropriately following the steps of an outbreak investigation | 5 | 5 | 4 | Accepted |
| 92 | Graduates implement or support the implementation of appropriate control measures during outbreak investigations | 4 | 5 | 4 | Accepted |
| 93 | Graduates use evidence-based decision-making processes in the workplace | 4 | 5 | 4 | Accepted |
| 94 | Graduates are developing or contributing to the development of public health policy | 4 | 5 | 4 | Accepted |
| 95 | Graduates are developing or contributing to the development of improved public health programs | 4.5 | 5 | 4 | Accepted |
| 96 | Graduates are contributing to improvements in work-related processes or procedures in their workplace | 4 | 5 | 4 | Accepted |
| 97 | Graduates are recognized, utilized and have influence in the workplace | 4 | 5 | 3 | Undetermined |
| 98 | Graduates transfer field epidemiology knowledge and skills to others through training and mentoring | 4 | 5 | 4 | Accepted |
| 99 | Graduates apply field epidemiology knowledge and skills to other fields | 4 | 5 | 4 | Accepted |
| 100 | Graduates contribute to raising the profile of field epidemiology, surveillance and/or public health | 4 | 4.25 | 4 | Accepted |
| 101 | Graduates report unintended positive and/or negative consequences of FETP (e.g. graduate received promotion due to new skills (positive consequence); graduate given more responsibilities with no increase in pay (negative consequences), etc) | 5 | 5 | 4 | Accepted |
| 102 | Graduates actively engage with and contribute to an FETP alumni network | 4 | 5 | 3 | Undetermined |
| 103 | Graduates develop and engage in networks and partnership to improve public health practice | 4 | 5 | 3 | Undetermined |
| 104 | Graduates share field epidemiology learnings and good practices with public health colleagues | 4 | 5 | 4 | Accepted |
| 105 | Established career pathway for graduates | 5 | 5 | 4 | Accepted |
| 106 | Graduates are in public health leadership roles across all tiers of the health system | 5 | 5 | 4 | Accepted |
| 107 | Key surveillance and disease control positions at all tiers of the health system are occupied by FETP graduates | 4 | 5 | 4 | Accepted |
| 108 | Skills of graduates are maintained and continually applied | 4 | 5 | 4 | Accepted |
| 109 | Decision makers confident in and engaged with FETP graduates and the services they provide | 4.5 | 5 | 4 | Accepted |
| 110 | Graduates develop and deliver field epidemiology related training activities to workplace colleagues | 4 | 5 | 4 | Accepted |
| 111 | Graduates are public health influencers in their workplace and the communities/populations they serve | 4 | 5 | 4 | Accepted |
| 112 | Graduates routinely conduct field projects/operational research to understand and address key public health challenges | 4 | 4 | 4 | Accepted |
| 113 | Graduates routinely design and implement public health interventions to address public health challenges | 4 | 5 | 4 | Accepted |
| 114 | Graduates effectively engage with communities when planning and delivering public health programs | 4 | 5 | 4 | Accepted |
| 115 | Decision makers utilize the evidence generated by graduates to improve public health programming | 4 | 5 | 4 | Accepted |
| 116 | Graduates are routinely using evidence-based decision making to inform guidelines, policy & programmatic activities | 4 | 5 | 4 | Accepted |
| 117 | Graduates are driving innovation and service improvements | 4 | 4 | 4 | Accepted |
| 118 | Graduates contribute to improved surveillance resulting in improved public health programming | 4 | 5 | 4 | Accepted |
| 119 | Graduates contribute to improved surveillance resulting in improved outbreak detection and response | 4 | 5 | 4 | Accepted |
| 120 | Graduates provide a response-ready workforce for outbreak and public health emergency response activities nationally and/or internationally | 5 | 5 | 4 | Accepted |
| 121 | Graduates effectively engage with communities/general public when investigating outbreaks and responding to public health threats | 4 | 5 | 4 | Accepted |
| 122 | Established pathway for FETP graduates to become FETP trainers, mentors, and program staff | 5 | 5 | 4 | Accepted |
| 123 | Graduates contribute to the national FETP(s) as trainers, mentors, and program staff | 5 | 5 | 4 | Accepted |
| 124 | Graduates contribute to improved access to public health services | 4 | 4.25 | 3 | Undetermined |
| 125 | Graduates contribute to improved quality of public health services | 4 | 5 | 3 | Undetermined |
| 126 | Graduates contribute to improve public health key performance indicators (KPIs), such as vaccine coverage, supervised deliveries, etc | 4 | 5 | 4 | Accepted |
| 127 | Graduates contribute to improved community/general public engagement resulting in community involvement in public health decision making processes affecting them | 4 | 5 | 3 | Undetermined |
| 128 | Graduates contribute to improved health literacy within community/general public | 4 | 5 | 3 | Undetermined |
| **No.** | **IMPACT INDICATORS - ROUND ONE** | **Median** | **Upper** | **Lower** | **Status** |
| 129 | FETP faculty and graduates contribute to the generation of public health intelligence and evidence-based recommendations to improve public health | 4 | 5 | 4 | Accepted |
| 130 | FETP faculty and graduates contribute to an evidence-based decision-making culture that drives public health programming and practice | 4 | 5 | 4 | Accepted |
| 131 | FETP faculty and graduates contribute to the development of evidence based public health policies and practices that are accepted, resourced, and implemented | 4 | 5 | 4 | Accepted |
| 132 | FETP faculty and graduates contribute to strong disease surveillance systems that guide public health programming and consistently supports the early detection & response to public health threats | 4 | 5 | 4 | Accepted |
| 133 | FETP faculty and graduates are influential in advocating for political and financial support for public health at all levels of the health system | 4 | 5 | 4 | Accepted |
| 134 | FETP faculty and graduates contribute to a culture of respectful community engagement when designing and delivering public health programs and response activities | 4 | 5 | 4 | Accepted |
| 135 | FETP is institutionalized, adequately funded and nationally recognised as an important public health workforce development program | 5 | 5 | 4 | Accepted |
| 136 | FETP is run by national staff without the need for external support | 5 | 5 | 4 | Accepted |
| 137 | FETP program is influential across public health networks nationally and internationally | 4.5 | 5 | 4 | Accepted |
| 138 | FETP faculty and graduates contribute to reduced outbreak related morbidity and mortality through timely and effective outbreak response activities | 4 | 5 | 4 | Accepted |
| 139 | FETP faculty and graduates contribute to reduced morbidity and mortality through improved access to and provision of public health services | 4 | 5 | 4 | Accepted |
| 140 | FETP faculty and graduates contribute to reduced morbidity and mortality through improved public health program design and delivery | 4 | 5 | 4 | Accepted |

| **No.** | **OUTPUT INDICATORS - ROUND TWO** | **Median** | **Upper** | **Lower** | **Status** |
| --- | --- | --- | --- | --- | --- |
| 6 | Number/percentage of trainees reporting that training is relevant, engaging, and useful | 4 | 4 | 3 | Undetermined |
| 10 | Approximate number of hours of direct one-on-one mentoring provided to trainees | 4 | 4 | 3 | Undetermined |
| New-1 | Number/percentage of trainees reporting satisfactory (or higher) mentoring experience | 4 | 4 | 3 | Undetermined |
| 17 | Number of deployments involving trainees outside of the country of their training (international deployments) [Number/percentage of trainees deployed for a response outside of the country of their training] | 3.5 | 4.25 | 2.75 | Undetermined |
| 18 | Number/percentage of deployed trainees who undertake post-deployment debriefs | 4 | 4 | 2.75 | Undetermined |
| 21 | Number of public health policy briefs written by trainees [﻿Number/percentage of trainees writing policy briefs] | 4 | 4 | 3 | Undetermined |
| 22 | Number of public health policies updated or developed by trainees [﻿Number/percentage of trainees updating or developing policies] | 3 | 4 | 3 | Undetermined |
| 24 | Number of papers published with trainees as lead authors [Number/percentage of trainees publishing papers as lead authors] | 4 | 5 | 3.75 | Undetermined |
| 25 | Number of papers published with trainees as co-authors [Number/percentage of trainees publishing papers as co-authors] | 4 | 4 | 3.75 | Undetermined |
| 30 | Number of work-related processes, policies or guidance documents updated or developed by trainees [Number/percentage of trainees updating or developing work-related processes, policies or guidance documents] | 4 | 4 | 3 | Undetermined |
| New-2 | Number/percentage of graduates reporting that the knowledge and skills from FETP is relevant for their current role | 4 | 5 | 4 | Accepted |
| 33 | Number/percentage of graduates completing higher level training / professional development (course name and level, e.g. leadership training, Master of Public Health, PhD, etc) | 3.5 | 4.25 | 2.75 | Undetermined |
| 36 | Number of field projects or operational research studies completed by graduates ﻿[Number/percentage of graduates completing field projects or operational research studies] | 4 | 4 | 3 | Undetermined |
| 41 | Number of deployments involving graduates outside of the country where they work (international deployment) /Number/percentage of trainees deployed for a response outside of the country where they work | 4 | 4 | 3 | Undetermined |
| 42 | Number/percentage of deployed graduates who undertake post-deployment debriefs | 3 | 4 | 2.75 | Undetermined |
| 45 | Number/percentage of graduates reporting that the development of policy briefs is part of their current role | 4 | 4 | 3 | Undetermined |
| 47 | Number of papers published with graduates as lead authors [Number/percentage of graduates publishing papers as lead authors] | 4 | 5 | 3.75 | Undetermined |
| 48 | Number of papers published with graduates as co-authors [Number/percentage of graduates publishing papers as co-authors] | 4 | 4 | 3.75 | Undetermined |
| 49 | Number of field epidemiology related conference abstracts submitted by graduates being accepted [﻿Number/percentage of graduates having field epidemiology related conference abstracts accepted] | 4 | 4 | 4 | Accepted |
| 50 | Number of FETP related presentations given by graduates at national, regional, international meetings or conferences [Number/ percentage of graduates giving FETP related presentations at national, regional, international meetings or conferences] | 4 | 4.25 | 3 | Undetermined |
| 51 | Number/percentage of graduates reporting that updating or developing work-related processes, policies or guidance documents is part of their current role | 4 | 4.5 | 3 | Undetermined |
| 55 | Number/percentage of graduates who are members of national, regional or international public health committees or working groups | 4 | 4.25 | 3 | Undetermined |
| 58 | Number of disease surveillance systems strengthened / key performance indicators improved due to inputs or contributions from graduates | 5 | 5 | 4 | Accepted |
| 61 | Number/percentage of communicable disease outbreak investigated within 24, 48 and 72 hours of being reported due to inputs or contributions from graduates | 4 | 4.25 | 4 | Accepted |
| 62 | Number/percentage of communicable disease outbreaks that meet 7-1-7 targets due to inputs or contributions from graduates | 4 | 4 | 4 | Accepted |
| **No.** | **OUTCOME INDICATORS - ROUND TWO** | **Median** | **Upper** | **Lower** | **Status** |
| 97 | Graduates are recognized, utilized and have influence in the workplace | 5 | 5 | 4 | Accepted |
| 102 | Graduates actively engage with and contribute to an FETP alumni network | 5 | 5 | 3.75 | Undetermined |
| 103 | Graduates develop and engage in networks and partnership to improve public health practice | 5 | 5 | 3 | Undetermined |
| 124 | Graduates contribute to improved access to public health services | 4 | 4 | 3 | Undetermined |
| 125 | Graduates contribute to improved quality of public health services | 4 | 5 | 3 | Undetermined |
| 127 | Graduates contribute to improved community/general public engagement resulting in community involvement in public health decision making processes affecting them | 4 | 4.25 | 4 | Accepted |
| 128 | Graduates contribute to improved health literacy within community/general public | 4 | 4 | 4 | Accepted |

**Additional file 3.** Comments from panellists and actions taken.

| **No.** | **OUTPUT INDICATORS** | **Comments from Panel** | **Action** |
| --- | --- | --- | --- |
| 2 | Number/percentage of trainees enrolled in training by role, position, and workplace location | Add clarity on what is meant by role and position type | Footnote added to evaluation framework: "Position descriptions (e.g. disease control officer, surveillance officer, etc) will vary from country to country; workplace location specifies the level of employment (e.g. frontline, district, regional, provincial, state, national)" |
| 3 | Number/percentage of trainees linked with public health mentors/supervisors | This should be 100%, if not, prompt remediation is required | Footnote added to evaluation framework: "this should be 100%, if not, remedial action is required" |
| 5 | Total length of time in the field/workplace (in weeks) | Include reference to FETP minimum standards which indicate the minimum program length and minimum time in the field | Footnote added to evaluation framework: "When assessing this indicator, evaluators should cross reference FETP minimum standards for accreditation. Currently, standards are available for advanced programs; programs need to be a minimum of 21 months in length with a min of 68 weeks engaged in epidemiologic practice" |
| 6 | Number/percentage of trainees reporting enjoyable learning experience | Subjective indicator; needs clarity; learning does not always need to be enjoyable; recommend rewording indicator; combine this indicator with the following one | Indicator combined with indicator below: "Number/percentage of trainees reporting that training is relevant, engaging, and useful"; Footnote added to evaluation framework: "It is generally regarded that learner satisfaction is correlated with learning outcomes" |
| 7 | Number/percentage of trainees reporting training relevance to current role | This seems like an important question for graduates but not while in training. For me, I want to know if the training is relevant to the graduates position AFTER training. I hear too many stories about people going through FETP training and then returning to clinical roles where they are NOT using any of their epi skills; combine this indicator with the previous one | Indicator combined with indicator above; also, a new indicator added for consideration in round 2: "Number/percentage of graduates reporting that the knowledge and skills from FETP are relevant for their current role" |
| 8 | Number/percentage of trainees reporting improved field knowledge and skills in key field epidemiology competencies | In general or in relation to specific competencies?; which competencies? | Footnote added to evaluation framework: "FETPs may have different competencies depending on the type of FETP (frontline, intermediate, advanced) and the priorities of the program" |
| 10 | Approximate number of hours of direct one-on-one mentoring provided to trainees | What's the operational definition of mentorship? Informal mentoring may be difficult to estimate/quantify; time spent does not always equate to quality | Footnote added to evaluation framework: "Mentoring includes any guidance provided to trainees by FETP faculty, support staff, or placement site supervisors; mentoring can be conducted in-person or remotely via email, phone, zoom": New indicator added for consideration in round 2 "Number/percentage of trainees reporting satisfactory (or higher) mentoring experience" |
| 11 | Number of operational research studies completed by trainees [Number/percentage of trainees completing operational research studies] | I am not sure what exactly is meant by operational research studies; does this includes field projects; not relevant for all levels of training | Indicator updated to read: "Number of field projects or operational research studies completed by trainees [Number/percentage of trainees completing field projects or operational research studies]": Asterisk added to clarify that this specific indicator it may not be relevant or expected for some FETPs or their graduates |
| 12 | Number of surveillance systems evaluated by trainees [Number/percentage of trainees evaluating surveillance systems] | Number of surveillance systems evaluated may not be an important indicator to include for standard assessment. However, the number/percentage of trainees evaluating surveillance systems can provide important process information about the implementation of the training program; not relevant for all levels of training | Asterisk added to clarify that this specific indicator it may not be relevant or expected for some FETPs or their graduates |
| 13 | Number of surveillance systems designed and established by trainees | This is depending on level of FETP tiers. It should be specified to what tiers will this indicator fitted well, intermediate or advanced. | Asterisk added to clarify that this specific indicator it may not be relevant or expected for some FETPs or their graduates |
| 14 | Number of outbreak investigations led by trainees [Number/percentage of trainees leading outbreak investigations] | Suggest re-considering "leading an outbreak" as for frontline, it is not expected from them to lead; not relevant for all levels of training | Asterisk added to clarify that this specific indicator it may not be relevant or expected for some FETPs or their graduates |
| 16 | Number of national mobilizations involving trainees [Number/percentage of trainees mobilized nationally] | Add clarity on what is meant by national mobilizations; important to include, but not relevant for all levels of training | Indicator reworded for clarity: "Number of national deployments involving trainees within the country of their training (national deployment) [Number/percentage of trainees deployed for a response within the country of their training]"; Asterisk added to clarify that this specific indicator it may not be relevant or expected for some FETPs or their graduates |
| 17 | Number of international mobilizations involving trainees [Number/percentage of trainees mobilized internationally] | Add clarity on what is meant by international mobilizations; important to include, but not relevant for all levels of training | Indicator reworded for clarity: "Number of deployments involving trainees outside of the country of their training (international deployment) [Number/percentage of trainees deployed for a response outside of the country of their training]: Asterisk added to clarify that this specific indicator it may not be relevant or expected for some FETPs or their graduates |
| 18 | Number/percentage of deployed trainees who undertake post-deployment debriefs | Add clarity on what is meant by and what is included in a post-deployment debrief. | Footnote added to evaluation framework: "Post-deployment debriefs are structured meetings to review the things that went well and did not go well, with the objective of improving for future deployments." |
| 21 | Number of policy briefs written by trainees [Number/percentage of trainees writing policy briefs] | Important to include, but not relevant for all levels of training | Asterisk added to clarify that this specific indicator it may not be relevant or expected for some FETPs or their graduates |
| 22 | Number of policies updated or developed by trainees [Number/percentage of trainees updating or developing policies] | Important to include, but not relevant for all levels of training | Asterisk added to clarify that this specific indicator it may not be relevant or expected for some FETPs or their graduates |
| 23 | Number of recommendations made by trainees that were implemented [Number/percentage of trainees making health program recommendations that were implemented] | Add clarity of what is meant by a trainee's recommendations; Agree this is interesting; implementation may occur months or years after fellow is finished or may contribute to it but the evidence may be combined with other evidence | Footnote added to evaluation framework: "Recommendations arising from trainee’s field epidemiology work, including surveillance, outbreak response, field projects, operational research, etc" |
| 24 | Number of papers published with trainees as lead authors [Number/percentage of trainees publishing papers as lead authors] | Would this be any publication or publication on the work conducted during FETP?: Important to include, but not relevant for all levels of training | Indicator reworded for clarity: "Number of FETP-related papers published with trainees as lead authors [Number/percentage of trainees publishing FETP-related papers as lead authors]"; Asterisk added to clarify that this specific indicator it may not be relevant or expected for some FETPs or their graduates |
| 25 | Number of papers published with trainees as co-authors [Number/percentage of trainees publishing papers as co-authors] | Would this be any publication or publication on the work conducted during FETP?: Important to include, but not relevant for all levels of training | Indicator reworded for clarity: "Number of FETP-related papers published with trainees as co-authors [Number/percentage of trainees publishing FETP-related papers as co-authors]": Asterisk added to clarify that this specific indicator it may not be relevant or expected for some FETPs or their graduates |
| 29 | Number of conference presentations given by trainees [Number/percentage of trainees giving conference presentations] | This indicator is captured in an earlier one; suggest changing this indicator to focus on formal or informal presentations given within the workplace | This indicator is a duplicate and is captured with an earlier indicator; New indicator added for consideration in round 2: "Number of FETP-related presentations given by trainees within their workplace or placement site [Number/percentage of trainees giving FETP-related presentations within their workplace or placement site]" |
| 35 | Number/percentage of graduates receiving a promotion within 12 months of graduating | Could be 2-3 years rather than 12 months; also add that this promotion due to FETP training | Indicator reworded for clarity: "Number/percentage of graduates receiving a promotion, attributed partially or entirely to their field epidemiology training, within 12, 24, and/or 36 months of graduating" |
| 36 | Number of operational research studies completed by graduates [Number/percentage of graduates completing operational research studies] | Since when?; Operational research should be expanded to include field projects; Important to include, but not relevant for all levels of training | Indicator reworded for clarity: "Number of field projects or operational research studies completed by graduates since finishing their field epidemiology training [Number/percentage of graduates completing field projects or operational research studies since finishing their field epidemiology training]"; Asterisk added to clarify that this specific indicator it may not be relevant or expected for some FETPs or their graduates |
| 37 | Number of surveillance systems evaluated by graduates [Number/percentage of graduates evaluating surveillance systems] | Since when? Important to include, but not relevant for all levels of training | Indicator reworded for clarity: "Number of surveillance systems evaluated by graduates since finishing their field epidemiology training [Number/percentage of graduates evaluating surveillance systems]"; Asterisk added to clarify that this specific indicator it may not be relevant or expected for some FETPs or their graduates |
| 40 | Number of national mobilizations involving graduates [Number/percentage of graduates mobilized nationally] | Add clarity on what is meant by national mobilizations; important to include, but not relevant for all levels of training | Indicator reworded for clarity: "Number of deployments involving graduates within the country where they work (national deployment) [Number/percentage of trainees deployed for a response within the country where they work]"; Asterisk added to clarify that this specific indicator it may not be relevant or expected for some FETPs or their graduates |
| 41 | Number of international mobilizations involving graduates [Number/percentage of graduates mobilized internationally] | Add clarity on what is meant by international mobilizations; important to include, but not relevant for all levels of training | Indicator reworded for clarity: "Number of deployments involving graduates outside of the country where they work (international deployment) [Number/percentage of trainees deployed for a response outside of the country where they work]"; Asterisk added to clarify that this specific indicator it may not be relevant or expected for some FETPs or their graduates |
| 42 | Number/percentage of deployed graduates who undertake post-deployment debriefs | Please specify what debriefs are for, what they entail | Footnote added to evaluation framework: "Post-deployment debriefs are structured meetings to review the things that went well and did not go well, with the objective of improving for future deployments" |
| 49 | Number of conference abstracts submitted by graduates being accepted [Number/percentage of graduates having conference abstracts accepted] | For what type of conference | Indicator reworded for clarity: "Number of field epidemiology related conference abstracts submitted by graduates being accepted /Number/percentage of graduates having field epidemiology related conference abstracts accepted" |
| 50 | Number of conference presentations given by graduates [Number/percentage of graduates giving conference presentations] | Clarify to include align with trainee's indicator; Agree, but more details on the statement is needed, presentation on what and for what? | Indicator reworded for clarity: "Number of FETP related presentations given by graduates at national, regional, international meetings or conferences /Number/ percentage of graduates giving FETP related presentations at national, regional, international meetings or conferences" |
| 53 | Number/percentage of [districts /provinces] in country with graduates | Include regions and states | Indicator reworded for clarity: "Number/percentage of districts, provinces, states and or regions in country with graduates" |
| 54 | Number/percentage of graduates employed by role, position, workplace type and location | Clarify “workplace type” | Footnote added to evaluation framework: "Workplace type may include government, non-government, international organization, etc." |
| 56 | Number of disease surveillance systems strengthened by trainees and graduates | This would not be easy to assess since it is not the sole task of a person; Consider in place of "strengthened" using "supported" by trainees/graduates. | Indicator reworded for clarity: "Number of disease surveillance systems supported by trainees and graduates" |
| 57 | Number of outbreak response systems and practices strengthened by trainees and graduates | Consider in place of "strengthened" using "supported" by trainees/graduates. | Indicator reworded for clarity: "Number of outbreak response systems supported by trainees and graduates" |
| 58 | Number of disease surveillance systems strengthened / key performance indicators improved | Do we have more specific term to replace "strengthen"?; specify due to the contributions of graduates | Indicator reworded for clarity: "Number of disease surveillance systems strengthened / key performance indicators improved due to inputs or contributions from graduates" |
| 59 | Number/percentage of graduate workplaces reporting improvements in surveillance | Add “because of input or contributions from FETP graduate(s)” | Indicator reworded for clarity: "Number/percentage of graduate workplaces reporting improvements in surveillance due to contributions from FETP graduate(s)" |
| 60 | Number/percentage of graduate workplaces reporting improvements in outbreak response | Add “because of input or contributions from FETP graduate(s)” | Indicator reworded for clarity: "Number/percentage of graduate workplaces reporting improvements in outbreak response due to contributions from FETP graduate(s)" |
| 61 | Number/percentage of communicable disease outbreaks where an investigation commenced within 24, 48 and 72 hours | Add “because of input or contributions from FETP graduate(s)” | Indicator reworded for clarity: "Number/percentage of communicable disease outbreak investigated within 24, 48 and 72 hours of being reported due to inputs or contributions from graduates" |
| 62 | Number/percentage of communicable disease outbreaks meeting 7-1-7 targets | Add “because of input or contributions from FETP graduate(s)”; Define 7-1-7 | Indicator reworded for clarity: "Number/percentage of communicable disease outbreaks that meet 7-1-7 targets due to inputs or contributions from graduates"; Footnote added to evaluation framework: "7-1-7 targets include: 7 days to detect a suspected infectious disease outbreak, 1 day to notify public health authorities to start an investigation and 7 days to complete an initial response)" |
| 63 | Number/percentage of evidence-based recommendations, developed by trainees and graduates, that have been implemented | Does this include evidence-based recommendations during outbreaks as well as field investigations. | Footnote added to evaluation framework: "Includes recommendation arising from outbreak investigations, surveillance evaluations, field projects, operational research, data analysis projects, etc." |
| 69 | FETP steering committee is established and functional | Not sure what the steering committee does or what is meant by functional | Footnote added to evaluation framework: "A steering committee is usually comprised of senior level managers and executives who provide advice and overall direction to the FETP." |
| 74 | Number of community/general public engagement activities conducted by trainees or graduates | Define what is included in community engagement | Footnote added to evaluation framework: "Activities that are focused on developing relationships with community or general public and seeking their input into or compliance with public health measures" |

| **No.** | **OUTCOME INDICATORS** |  |  |
| --- | --- | --- | --- |
| 78 | Trainees undertake field projects, such as surveillance evaluations, operational research, or interventions | Not relevant for all levels of training | Asterisk added to clarify that this specific indicator it may not be relevant or expected for some FETPs or their graduates |
| 80 | Trainees improve a surveillance system or component of a surveillance system | Not relevant for all levels of training | Asterisk added to clarify that this specific indicator it may not be relevant or expected for some FETPs or their graduates |
| 88 | Graduates undertake field projects, such as surveillance evaluations, operational research, or interventions to improve health systems or health outcomes | Redundant or overlaps with previous indicator | Footnote added to evaluation framework: "This outcome indicator is similar to its companion output indictor; evaluators may wish to choose either the output or the outcome indicator; typically, outcome indictors go beyond ‘counting’ and include descriptions or additional details." |
| 89 | Graduates improve surveillance systems or components of a surveillance system | Redundant or overlaps with previous indicator | Footnote added to evaluation framework: "This outcome indicator is similar to its companion output indictor; evaluators may wish to choose either the output or the outcome indicator; typically, outcome indictors go beyond ‘counting’ and include descriptions or additional details." |
| 97 | Graduates are recognized, utilized and have influence in the workplace | In terms of what? not clear | Footnote added to evaluation framework: "Graduates are recognised and utilised for field epidemiology related activities by management within the workplace; the results of their work are highly regarded by decision makers" |
| 99 | Graduates apply field epidemiology knowledge and skills to other fields | Does it include programs / academics/ research?; What do you mean? Please provide examples | Footnote added to evaluation framework: "Other fields include anything that is not related to field epidemiology or public health, such as scientistic research, management, clinical practice, etc" |
| 100 | Graduates contribute to raising the profile of field epidemiology, surveillance and/or public health | What is included in “raising profile of field epidemiology”? | Footnote added to evaluation framework: "Includes recognition by management of the value field epidemiologists bring to the organisation" |
| 108 | Skills of graduates are maintained and continually applied | Not sure what “continually applied” means | Indicator reworded for clarity: "Field epidemiology related skills of graduates are maintained and routinely applied in their work" |
| 109 | Decision makers confident in and engaged with FETP graduates and the services they provide | Unclear who the decision makers are | Footnote added to evaluation framework: "Decision makers include managers, policy makers, executives etc within the graduates workplace or other relevant individuals utilizing the services of the graduate." |
| 111 | Graduates are public health influencers in their workplace and the communities/populations they serve | I am not sure what “influencer” means here | Indicator reworded for clarity: "Graduates are influential in their workplace and/or the communities and populations they serve" |
| 112 | Graduates routinely conduct field projects/operational research to understand and address key public health challenges | “Routinely”, this is vague and hard to assess. | Indicator reworded for clarity: "Graduates conduct field projects/operational research to understand and address key public health challenges" |
| 113 | Graduates routinely design and implement public health interventions to address public health challenges | “Routinely”, this is vague and hard to assess. | Indicator reworded for clarity: "Graduates design and implement public health interventions to address public health challenges " |
| 114 | Graduates effectively engage with communities when planning and delivering public health programs | “Effectively”, this is vague and hard to assess. | Indicator reworded for clarity: "Graduates engage with communities when planning and delivering public health programs" |

**Additional file 4.** Final impact evaluation framework for Field Epidemiology Training Programs

**FETP Impact Evaluation Framework for Field Epidemiology Training Programs**

Indicators without italics are considered high-priority indicators for consideration in the evaluation of standard (non-speciality track) Frontline, Intermediate and Advanced FETPs.

Indicators in *italics* are considered lower-priority indicators for consideration in the evaluation of standard (non-speciality track) Frontline, Intermediate and Advanced FETPs.

Indicators marked with a ‘*‘ may not be relevant or expected for some FETPs or their graduates.

| **LEVEL OF CHANGE** | **ACTIVITY** | **OUTPUTS**  *Products, projects, or activities which result from the training program.*  **TRAINEES: outputs during the training program (typically things that can be counted)** | **OUTCOMES**  *Short-term and medium-term effects of the training program*  **TRAINEES: short-term outcomes achieved by trainees during training** | **IMPACT​**  *Longer-term effects produced by the training program.*  **TRAINEES: not relevant** |
| --- | --- | --- | --- | --- |
| **Trainees**​ | **FIELD EPIDEMIOLOGY TRAINING** providing knowledge and skills | **Trainees participate in a competency-based Field Epidemiology Training Program and apply skills and knowledge.**  **Training Participation**   - Gender breakdown of trainees - Number/percentage of trainees enrolled in training by position and workplace location ^[[1]](#footnote-2)^ - Number/percentage of trainees linked with public health mentors/supervisors ^[[2]](#footnote-3)^   **Training Quality and Relevance**   - Number of hours of training in a classroom-type setting (including virtual training) - Total length of time in the field/workplace (in weeks) ^[[3]](#footnote-4)^ - Number/percentage of trainees reporting that training is relevant, engaging, and useful ^[[4]](#footnote-5)^ - Number/percentage of trainees reporting improved knowledge and skills in field epidemiology competencies ^[[5]](#footnote-6)^ - Number/percentage of trainees that meet the program’s core competencies - Approximate number of hours of direct mentoring provided to trainees each month ^[[6]](#footnote-7)^ - Number/percentage of trainees reporting satisfactory (or higher) mentoring experience   **Operational Research, Surveillance and Outbreak Investigation**   - Number of field projects or operational research studies completed by trainees [Number/percentage of trainees completing field projects or operational research studies] * - Number of surveillance systems evaluated by trainees [Number/percentage of trainees evaluating surveillance systems] * - Number of surveillance systems designed and established by trainee * - Number of outbreak investigations led by trainees [Number/percentage of trainees leading outbreak investigations] * - Number of outbreak investigations supported by trainees [Number/percentage of trainees supporting outbreak investigations] - Number of deployments involving trainees within the country of their training (national deployment) [Number/percentage of trainees deployed for a response within the country of their training] * - *Number of deployments involving trainees outside of the country of their training (international deployment) [Number/percentage of trainees* deployed for a response outside of the country of their training*]* * - *Number/percentage of deployed trainees who undertake post-deployment debriefs by the FETP ^[[7]](#footnote-8)^** - Number/percentage of trainee mobilisations meeting the needs of the person or organisation requesting FETP support for a response *   **Written Products, Presentations and Processes**   - Number of epidemiological reports prepared by trainees /Number/percentage of trainees preparing epidemiological reports - Number of recommendations made by trainees that were implemented / Number/percentage of trainees making health program recommendations that were implemented ^[[8]](#footnote-9)^ - *Number of FETP-related papers published with trainees as lead authors [Number/percentage of trainees publishing FETP-related papers as lead authors]* * - *Number of FETP-related papers published with trainees as co-authors [Number/percentage of trainees publishing FETP-related papers as co-authors]* * - Number of FETP-related presentations given by trainees within their workplace/placement site [Number/percentage of trainees giving FETP-related presentations within their workplace or placement site] ^[[9]](#footnote-10)^ - Number of FETP-related presentations given by trainees at national, regional, international meetings or conferences [Number/ percentage of trainees giving FETP-related presentations at national, regional, international meetings or conferences] - Number of FETP-related conference abstracts submitted by trainees being accepted [Number/percentage of trainees having FETP-related conference abstracts accepted] * - *Number of work-related processes or guidance documents updated or developed by trainees [Number/percentage of trainees updating or developing work-related processes or guidance documents]* | **Trainees are competent and committed to applying their skills and knowledge in their workplace or placement site**  **Trained Field Epidemiologists**   - Trainees demonstrate the application of field epi competencies throughout the training - Trainees are confident in applying their knowledge and skills in their workplace / placement site - Trainees report unintended positive and/or negative consequences of FETP ^[[10]](#footnote-11)^   **Competent Field Epidemiologists**   - Trainees undertake field projects, such as surveillance evaluations, operational research, or interventions * - Trainees contribute to improvements in work-related processes, policies or guidance document at their workplace/placements site - Trainees improve a surveillance system or component of a surveillance system * - Trainees analyse and interpret surveillance data to inform decision making - Trainees investigate outbreaks by appropriately following the steps of an outbreak investigation - Trainees implement or support the implementation of appropriate control measures during outbreak investigations | ​ |

| **LEVEL OF CHANGE** | **ACTIVITY** | **OUTPUTS**  *Products, projects, or activities which result from the training program.*  **GRADUATES: individual outputs following graduation** | **OUTCOMES**  *Short-term and medium-term effects of the training program*  **GRADUATES: Short-term individual outcomes achieved by graduates** | **IMPACT​**  *Longer-term effects produced by the training program.*  **GRADUATES: not relevant** |
| --- | --- | --- | --- | --- |
| **Graduates**​ | **FETP GRADUATES CONTRIBUTING** across different levels of the public health system​ | **Graduates develop and apply skills to strengthen disease surveillance, investigate outbreaks, conduct operational research, and share findings through papers, reports, and presentations.**  **Graduates**   - Gender breakdown of graduates - Number/percentage of graduates completing FETP training by position and workplace location - Number/percentage of graduates reporting that the knowledge and skills from FETP are relevant for their current role - Number/percentage of graduates given additional responsibilities in their employment because of their field epidemiology training - Number/percentage of graduates receiving a promotion, attributed partially or entirely to their field epidemiology training, within 12, 24, and/or 36 months of graduating   **Operational Research, Surveillance and Outbreak Investigation**   - Number of field projects or operational research studies completed by graduates since finishing their field epidemiology training [Number/percentage of graduates completing field projects or operational research studies since finishing their field epidemiology training] * - Number of surveillance systems evaluated by graduates since finishing their field epidemiology training [Number/percentage of graduates evaluating surveillance systems since finishing their field epidemiology training] * - Number of outbreak investigations led by graduates since finishing their field epidemiology training [Number/percentage of graduates leading outbreak investigations since finishing their field epidemiology training] * - Number of outbreaks investigations supported by graduates since finishing their field epidemiology training [Number/percentage of graduates supporting outbreak investigations] - Number of deployments involving graduates within the country where they work (national deployment) [Number/percentage of trainees deployed for a response within the country where they work] * - *Number of deployments involving graduates outside of the country where they work (international deployment) [Number/percentage of trainees* deployed for a response outside of the country where they work*]* * - Number/percentage of graduates supporting an Incident Management System (IMS) during a public health emergency response   **Written products, Presentations and Processes**   - Number/percentage of graduates reporting that the development of epidemiological reports is part of their current role - *Number/percentage of graduates reporting that the development of policy briefs is part of their current role ** - Number/percentage of graduates reporting that improving public health programs is part of their current role - *Number of field epidemiology-related papers published with graduates as lead authors [Number/percentage of graduates publishing field epidemiology-related papers as lead authors]* - Number of field epidemiology-related papers published with graduates as co-authors [Number/percentage of graduates publishing field epidemiology-related papers as co-authors] - Number of field epidemiology-related conference abstracts submitted by graduates being accepted [Number/percentage of graduates having FETP-related conference abstracts accepted] - Number of field epidemiology-related presentations given by graduates at national, regional, international meetings or conferences [Number/ percentage of graduates giving FETP-related presentations at national, regional, international meetings or conferences] - *Number/percentage of graduates reporting that updating or developing work-related processes or guidance documents is part of their current role* | **Skilled graduates strengthen public health activities in their workplace and contribute to a community of practice through alumni networks.**  **Skilled Field Epi Workforce**   - Graduates employed in positions where field epi knowledge and skills are required - Graduates provided opportunities to apply skills and knowledge in the workplace - Graduates confident in applying knowledge and skills in the workplace - Graduates progress in their careers as a result of their graduation from a FETP   **Contributing Field Epi Graduates**   - Graduates undertake field projects, such as surveillance evaluations, operational research, or interventions to improve health systems or health outcomes ^[[11]](#footnote-12)^ - Graduates improve surveillance systems or components of a surveillance system ^[[12]](#footnote-13)^ - Graduates routinely analyse and interpret surveillance data to inform decision making - Graduates investigate outbreaks by appropriately following the steps of an outbreak investigation - Graduates implement or support the implementation of appropriate control measures during outbreak investigations - Graduates use evidence-based decision-making processes in the workplace - Graduates are developing or contributing to the development of public health policy - Graduates are developing or contributing to the development of improved public health programs - Graduates are contributing to improvements in processes or procedures in their workplace - Graduates are recognized, utilized and have influence in the workplace ^[[13]](#footnote-14)^ - Graduates transfer field epidemiology knowledge and skills to others through training and mentoring - Graduates apply field epidemiology knowledge and skills to other fields ^[[14]](#footnote-15)^ - Graduates contribute to raising the profile of field epidemiology, surveillance and/or public health in their workplace ^[[15]](#footnote-16)^ - Graduates report unintended positive and/or negative consequences of FETP^[[16]](#footnote-17)^   **Networks and Partnerships**   - *Graduates actively engage with and contribute to an FETP alumni network* - Graduates develop and engage in networks and partnerships to improve public health practice - Graduates share field epidemiology learnings and good practices with public health colleagues | ​  ​ |

| **LEVEL OF CHANGE** | **Activity​** | **Outputs​**  Products, projects or activities that result from the training program  **PUBLIC HEALTH SYSTEM: outputs that affect the public health system** | **Outcomes​**  Short-term and medium-term effects of the training program outputs  **PUBLIC HEALTH SYSTEM: short-term and medium-term effects on the public health system** | **IMPACT​**  Longer-term positive and negative, primary, and secondary long-term effects produced by the training program, directly or indirectly, intended or unintended.  **PUBLIC HEALTH SYSTEM: longer-term effects of the program on the public health system** |
| --- | --- | --- | --- | --- |
| **Public Health System​** | **HEALTH SYSTEMS STRENGTHENING** through the application of field epidemiology skills | **Graduates embedded across all levels of the public health system, conducting projects and activities that strengthen public health systems. Graduates become junior FETP staff.**  **Field Epidemiology Workforce**   - Number/percentage of graduates working across different tiers of the health system (e.g. district, provincial, and national levels) - Number/percentage of districts, provinces, states and or regions in the country with graduates - Number/percentage of graduates employed by role, position, workplace type and location ^[[17]](#footnote-18)^ - *Number/percentage of graduates who are members of national, regional or international public health committees or working groups*   **Support for Surveillance and Outbreak Response**   - Number of disease surveillance systems supported by trainees and graduates - Number of outbreak response systems supported by trainees and graduates - Number of disease surveillance systems strengthened / key performance indicators improved due to inputs or contributions from graduates - Number/percentage of graduate workplaces reporting improvements in surveillance due to contributions from FETP graduate(s) - Number/percentage of graduate workplaces reporting improvements in outbreak response due to contributions from FETP graduate(s) - Number/percentage of communicable disease outbreak investigated within 24, 48 and 72 hours of being reported due to inputs or contributions from graduates - Number/percentage of communicable disease outbreaks that meet 7-1-7 targets due to inputs or contributions from graduates ^[[18]](#footnote-19)^   **Support for Health Systems**   - Number/percentage of evidence-based recommendations, developed by trainees and graduates, that have been implemented ^[[19]](#footnote-20)^ - Number/percentage of work-related processes, policies or guidance documents developed by trainees and graduates that have been implemented - Number/percentage of placement sites/workplaces reporting improvement in health systems due to inputs or contributions from trainees/graduates (e.g. strengthened surveillance, outbreak response, program delivery, etc) ^[[20]](#footnote-21)^   **FETP Support**   - Number/percentage of FETP trainers and mentors who are graduates of the program - Number/percentage of FETP staff (e.g. Director, Convenor) who are graduates of the program - FETP is recognised in official governmental/institutional planning documents ^[[21]](#footnote-22)^ * - FETP steering committee is established and functional ^[[22]](#footnote-23)^ - Number/percentage of FETP positions fully staffed - Percentage of FETP budget funded by the national government - FETP accredited by TEPHINET * | **Field Epidemiology workforce contributes to strengthening the public health system through routine application of knowledge and skills. FETP graduates support FETP as trainers and mentors.**  **Influential Field Epi workforce**   - Established career pathway for graduates - Graduates are in public health leadership roles across all tiers of the health system - Key surveillance and disease control positions at all tiers of the health system are occupied by FETP graduates - Field epidemiology related skills of graduates are maintained and applied in their work - Decision makers confident in and engaged with FETP graduates and the services they provide ^[[23]](#footnote-24)^ - Graduates develop and deliver field epidemiology related training activities to workplace colleagues - Graduates are influential in their workplace and/or the communities and populations they serve   **Health Systems Strengthened**   - Graduates conduct field projects/operational research to understand and address key public health challenges - Graduates design and implement public health interventions to address public health challenges - Graduates engage with communities when planning and delivering public health programs - Decision makers utilize the evidence generated by graduates to improve public health programming - Graduates are routinely using evidence-based decision making to inform guidelines, policy & programmatic activities - Graduates are driving innovation and service improvements   **Stronger Surveillance and Outbreak Response**   - Graduates contribute to improved surveillance resulting in improved public health programming - Graduates contribute to improved surveillance resulting in improved outbreak detection and response - Graduates provide a response-ready workforce for outbreak and public health emergency response activities nationally and/or internationally - Graduates effectively engage with communities/general public when investigating outbreaks and responding to public health threats   **Graduate Support for FETP(s)**   - Established pathway for FETP graduates to become FETP trainers, mentors, and program staff - Graduates contribute to the national FETP(s) as trainers, mentors, program staff | **Strong public health systems across PNG. Strong and Sustainable FETP is established.**  **Strong Health Systems**   - FETP faculty and graduates contribute to the generation of public health intelligence and evidence-based recommendations to improve public health - FETP faculty and graduates contribute to an evidence-based decision-making culture that drives public health programming and practice - FETP faculty and graduates contribute to the development of evidence based public health policies and practices that are accepted, resourced, and implemented - FETP faculty and graduates contribute to strong disease surveillance systems that guide public health programming and consistently supports the early detection & response to public health threats - FETP faculty and graduates are influential in advocating for political and financial support for public health at all levels of the health system - FETP faculty and graduates contribute to a culture of respectful community engagement when designing and delivering public health programs and response activities   **Sustainable FETP(s)**   - FETP is institutionalized, adequately funded and nationally recognised as an important public health workforce development program - FETP is run by national staff without the need for external support - FETP program is influential across public health networks nationally and internationally |

| **LEVEL OF CHANGE** | **Activity​** | **Outputs​**  Products, projects or activities that result from the training program  **COMMUNITY: outputs that affect the community/general public** | **Outcomes​**  Short-term and medium-term effects of the training program outputs  **COMMUNITY: short term outcomes of the program affecting the community/general public** | **IMPACT​**  Longer-term positive and negative, primary and secondary long-term effects produced by the training program, directly or indirectly, intended or unintended.  **COMMUNITY: longer-term effects of the program on the community/general public** |
| --- | --- | --- | --- | --- |
| **Community​ / General Public** | **RESPONSIVE AND EFFECTIVE HEALTH SYSTEM ​**providing health services improving the health of the public | **Community/population-based public health activities and outreach programs conducted**   - Number of new or strengthened community/population-based public health programs conducted or coordinated by trainees or graduates - Number of community/public engagement activities conducted by trainees or graduates ^[[24]](#footnote-25)^ | **Improved access to higher quality public health services addressing priority community/general public needs; community/general public engaged in public health decision making.**   - Graduates contribute to improving public health key performance indicators (KPIs), such as vaccine coverage, supervised deliveries, etc - Graduates contribute to improved community/general public engagement resulting in community involvement in public health decision-making processes affecting them - Graduates contribute to improved health literacy within the community/general public | **Improved public health realized through reduced morbidity and mortality to communicable and non-communicable diseases.**   - FETP faculty and graduates contribute to reduced outbreak-related morbidity and mortality through timely and effective outbreak response activities - FETP faculty and graduates contribute to reduced morbidity and mortality through improved access to and provision of public health services - FETP faculty and graduates contribute to reduced morbidity and mortality through improved public health program design and delivery |

1. Position descriptions (e.g. disease control officer, surveillance officer, etc) will vary from country to country; workplace location specifies the level of employment (e.g. frontline, district, regional, provincial, state, national) [↑](#footnote-ref-2)
2. Should be 100%, if not, remedial action is required. [↑](#footnote-ref-3)
3. When assessing this indicator, evaluators should cross reference FETP [minimum standards for accreditation](https://www.tephinet.org/sites/default/files/ACCREDITATION%20OF%20FETPs%20MINIMUM%20INDICATORS%20AND%20STANDARDS_0.pdf). Currently standards are available for advanced programs; programs need to be a min 21 months in length with a min of 68 weeks engaged in epidemiologic practice. [↑](#footnote-ref-4)
4. It is generally regarded that learner satisfaction is correlated with achieving outcomes [↑](#footnote-ref-5)
5. FETPs may have different competencies depending on the type of FETP (frontline, intermediate, advanced) and the priorities of the program. [↑](#footnote-ref-6)
6. Mentoring includes any guidance provided to fellows by FETP faculty, support staff, or placement site supervisors; mentoring can be conducted in-person or remotely via email, phone, zoom. [↑](#footnote-ref-7)
7. Post-deployment debriefs are structured meetings conducted by the FETP to review the experience of the trainees while on deployment and to assess the things that went well and did not go well, with the objective of improving for future deployments. [↑](#footnote-ref-8)
8. Recommendations arising from trainee’s field epidemiology work, including surveillance, outbreak response, field projects, operational research, etc. [↑](#footnote-ref-9)
9. Includes formal and informal presentations to workplace colleagues, supervisors, managers, executive, etc. [↑](#footnote-ref-10)
10. For example, a trainee is given new work opportunity because of the skills they have acquired (positive consequence); or a trainee works overtime in order to manage both regular work and FETP assignments (negative consequences). [↑](#footnote-ref-11)
11. This outcome indicator is similar to its companion output indictor; evaluators may wish to choose either the output or the outcome indicator; typically, outcome indictors go beyond ‘counting’ and include descriptions or additional details. [↑](#footnote-ref-12)
12. As above [↑](#footnote-ref-13)
13. Graduates are recognised and utilised for field epidemiology related activities by management within the workplace; the results of their work are highly regarded by decision makers. [↑](#footnote-ref-14)
14. Other fields include anything that is not related to field epidemiology or public health, such as scientistic research, management, clinical practice, etc. [↑](#footnote-ref-15)
15. Includes recognition by management of the value field epidemiologists bring to the organisation. [↑](#footnote-ref-16)
16. For example, graduate received promotion due to new skills (positive consequence); graduate given more responsibilities with no increase in pay (negative consequences), etc) [↑](#footnote-ref-17)
17. Workplace type may include government, non-government, international organisation, etc. [↑](#footnote-ref-18)
18. 7-1-7 targets include: 7 days to detect a suspected infectious disease outbreak, 1 day to notify public health authorities to start an investigation and 7 days to complete an initial response). [↑](#footnote-ref-19)
19. Includes recommendation arising from outbreak investigations, surveillance evaluations, field projects, operational research, data analysis projects, etc. [↑](#footnote-ref-20)
20. Assess from the perspective of workplace supervisors, managers or other relevant individuals in the workplace. [↑](#footnote-ref-21)
21. Relevant for FETPs which are housed within national ministries of health. [↑](#footnote-ref-22)
22. A steering committee is usually comprised of senior level managers and executives who provide advice and overall direction to the FETP. [↑](#footnote-ref-23)
23. Decision makers include managers, policy makers, executives, etc, within the graduate’s workplace or other relevant individuals utilising the services of the graduate. [↑](#footnote-ref-24)
24. Activities that are focused on developing relationships with community or public and seeking their input into or compliance with public health measures. [↑](#footnote-ref-25)
